# Regulation mechanism of the long-chain *n*-alkane monooxygenase gene *almA* in *Acinetobacter venetianus* RAG-1

**DOI:** 10.1128/aem.02050-24

**Published:** 2024-12-26

**Authors:** Shuai Chen, Lu Cao, Tianhua Lv, Jia Liu, Ge Gao, Mingchang Li, Liyuan Sun, Wenzhuo Tian, Yutong Tian, Guoqiang Li, Ting Ma

**Affiliations:** 1 Key Laboratory of Molecular Microbiology and Technology, Ministry of Education, College of Life Sciences, Nankai University 117931 , Tianjin, Tianjin, China; 2 Tianjin Institute of Industrial Biotechnology, Chinese Academy of Sciences 165087 , Tianjin, Tianjin, China; 3 Tianjin Engineering Technology Center of Green Manufacturing Biobased Materials, Nankai University 12538 , Tianjin, China; Shanghai Jiao Tong University, Shanghai, China

**Keywords:** *Acinetobacter venetianus *RAG-1, long-chain *n*-alkanes, AlmA, AraC/XylS family transcriptional regulator, APR1

## Abstract

As toxic pollutants, *n*-alkanes are pervasively distributed in most environmental matrices. Although the alkane monooxygenase AlmA plays a critical role in the metabolic pathway of solid long-chain *n*-alkanes (≥C_20_) that are extremely difficult to degrade, the mechanism regulating this process remains unclear. Here, we characterized the function of AlmA in *Acinetobacter venetianus* RAG-1, which was mainly involved in the degradation of long-chain *n*-alkanes (C_26_–C_38_), among which, *n*-C_32_ induced the *almA* promoter activity most. APR1 (AlmA Positive Regulator) that it is an AraC/XylS-type transcription regulator, a potential transcriptional regulator of *almA*, was screened by DNA-pull down, which was determined by conserved domain analysis. The deletion of *apR1* severely reduced the capacity of strain RAG-1 to utilize long-chain *n*-alkanes (C_22_–C_38_), indicating the involvement of APR1 in *n*-alkanes degradation. The results of the APR1-dependent reporter system, electrophoretic mobility shift assay, and microscale thermophoresis further proved that APR1 was able to directly bind to the *almA* promoter region, thus activating the *almA* transcription. Furthermore, APR1 could inhibit self-expression through autoregulation in the absence of long-chain *n*-alkanes. *n*-C_32_ acted as a ligand of APR1, and the amino acid residues Val10, Gln50, Ala99, and Ile106 at the N-terminus of APR1 were necessary for binding *n*-C_32_. In addition, the key amino acid residues of APR1 within the C-terminal helix-turn-helix motif that bound to the downstream promoter region were confirmed by multiple sequence alignment and site-directed mutagenesis. The homologs of AlmA and APR1 shared a similar evolutionary course in the *Proteobacteria*; thus, this mode of regulation might be relatively conserved.

**IMPORTANCE:**

The extreme hydrophobicity of long-chain *n*-alkanes ({greater than or equal to}C_20_) presents a significant challenge to their degradation in natural environments. It is, therefore, imperative to elucidate the regulatory mechanisms of the metabolic pathways of long-chain *n*-alkanes, which will be of great significance for the future application of hydrocarbon-degrading bacteria to treat oil spills. However, the majority of current studies have focused on the regulatory mechanisms of short- and medium-chain *n*-alkanes, with long-chain *n*-alkanes receiving comparatively little attention. In this study, we identified APR1, a transcriptional regulator of the alkane monooxygenase AlmA in *Acinetobacter venetianus* RAG-1, and characterized its function and regulatory mechanism. In the presence of ligand *n*-C_32_, APR1 could directly activate the transcription of *almA*, which was involved in the *n*-C_32_ metabolism. The amino acid residue unique to the C-terminal DNA-binding domain of AraC/XylS type *n*-alkanes transcription regulators was also identified. These findings further improved our understanding of the long-chain *n*-alkanes degradation mechanism, which is important for the management of petroleum pollution.

## INTRODUCTION

Alkanes, a main constituent of petroleum, are saturated hydrocarbons consisting of carbon and hydrogen atoms, widely spread in the natural environment. Among them, long-chain *n*-alkanes (≥C_20_) are extremely hydrophobic and difficult to degrade (1, 2), Consequently, in the event of a petroleum spill, the release of significant quantities of alkanes would inflict considerable damage to the surrounding environment (3). Nevertheless, many microorganisms have been documented to metabolize alkanes, most of which are bacteria, such as *Acinetobacter* sp., *Pseudomonas* sp., and *Alcanivorax* sp. (4, 5). Among them, alkane hydroxylation is the initial and rate-limiting step in alkane catabolism (6), with alkane hydroxylases as the pivotal enzyme responsible for catalyzing this reaction (7). Up to now, numerous hydroxylases capable of oxidizing short- and medium-chain *n*-alkanes (<C_20_) (8) have been identified, including diiron/copper-containing methane monooxygenase enzymes (MMOs) (9, 10), cytochrome P450 enzymes (11), and the membrane-integrated nonheme iron monooxygenase AlkB (12, 13). However, the only hydroxylases responsible for the oxidation of long-chain *n*-alkanes (≥C_20_) are monooxygenases LadA and AlmA (14, 15). AlmA, a flavin adenine dinucleotide (FAD)-dependent monooxygenase, was first identified in *Acinetobacter* sp. strain DSM17874 (16), and its homologs are prevalent in *Acinetobacter* strains that are capable of degrading long-chain *n*-alkanes (17, 18). While the function of AlmA varies among strains, its homologs have been identified to possess terminal oxidation activity against *n*-alkanes (C_10_–C_36_) in *Alcanivorax dieselolei* B-5 (19) and Baeyer–Villiger oxidation activity against aliphatic 2-ketones (C_10_–C_16_) in *Acinetobacter baylyi* ADP1 (20). In our previous study, an *almA* gene was identified in *Acinetobacter venetianus* RAG-1, with the ability to degrade *n*-alkanes (C_10_–C_38_), and involved in the metabolic pathway of long-chain *n*-alkanes (C_22_–C_38_) (21).

Furthermore, corresponding alkane regulatory mechanisms exist in hydrocarbon-degrading bacteria. The currently reported transcriptional regulators mainly belong to the following families, namely, LuxR/MalT (22, 23), AraC/XylS (2, 24, 25), GntR (26), and TetR (27). In *Pseudomonas putida* GPo1, the AlkS of LuxR/MalT family activates *alkS* gene expression when induced by effectors C_5_–C_10_ *n*-alkanes and can reduce self-expression level by autoregulation in the absence of alkanes (28). Also, the AlkR of the AraC/XylS family activates *alkM* transcription in *Acinetobacter* sp. ADP1 and participates in *n*-alkanes (C_12_–C_18_) metabolic pathway (24). However, there is only one article on the regulation mechanism of *almA*. In *A. dieselolei* B5, it was demonstrated that the expression of *almA* is inhibited by the regulatory protein AlmR based on growth and qPCR experiments (19).

The AraC/XylS family is an important class of transcriptional regulators that are widely involved in the regulation of metabolic pathways in organisms (29). These members of the family usually contain two functional domains, the ligand-binding domain of the N-terminal region and the DNA-binding domain of the C-terminal region. The ligand-binding domain varies greatly among the members, whereas the DNA-binding domain is relatively conserved (30).

To date, no studies have been conducted examining the role of AraC/XylS family members in modulating *almA* expression. Here, APR1, an activator of *almA* in *A. venetianus* RAG-1, which participated in the regulation of the long-chain *n*-alkanes (C_22_–C_38_) metabolic pathway, was identified. In particular, we found that APR1 could activate the *almA* expression when induced by the ligand C_32_, and the C-terminal DNA-binding domain of *n*-alkane transcriptional regulators had a key amino acid residue that is unique among other AraC/XylS-type regulators. Furthermore, phylogenetic analysis indicated that this transcriptional regulatory mechanism may be widespread in other *n*-alkane-degrading bacteria of *Proteobacteria*.

## RESULTS

### Identification of AlmA, the key enzyme responsible for utilization of long-chain *n*-alkanes (C_26_–C_38_) in RAG-1

**Fig 1:**
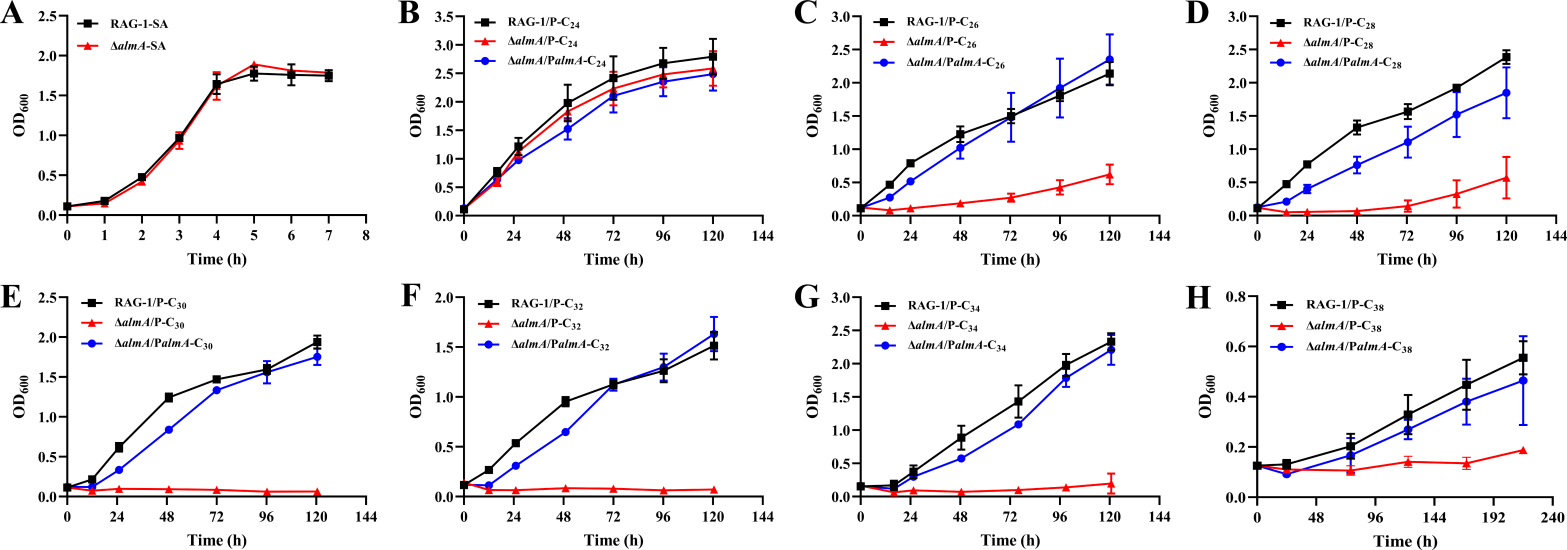
Effects of AlmA on various long-chain *n*-alkanes utilization. Growth curves of RAG-1/P (RAG-1 containing pMK vector), Δ*almA*/P (Δ*almA* containing pMK vector), and Δ*almA*/P*almA* (Δ*almA* containing *almA* complemented plasmid) strains cultured in basal salt medium (BSM) with sodium acetate (SA) (**A**), *n*-C_24_ (**B**), *n*-C_26_ (**C**), *n*-C_28_ (**D**), *n*-C_30_ (**E**), *n*-C_32_ (**F**), *n*-C_34_ (**G**), *n*-C_38_ (**H**) as the sole carbon source. Values are shown as means ± SD (*n* = 3).

In a previous study, we found that the F959_RS03485 gene (*almA*) was involved in the metabolism of long-chain *n*-alkanes (C_22_–C_38_) in RAG-1 by testing whether the single and double-deletion mutants grown on *n*-alkanes (21). To explore the function of AlmA in detail, its knockout (Δ*almA*/P) and complemented (Δ*almA*/P*almA*) strains were constructed, then the growth of mutants in basal salt medium (BSM) with different long-chain *n*-alkanes (≥C_24_) as the sole carbon source were analyzed. The results showed that the deletion of *almA* had no significant effect on growth in lysogeny broth (LB) medium, BSM with sodium acetate (SA), and C_24_ as the sole carbon source (Fig. S1; Fig. 1A and B). However, the Δ*almA*/P mutant completely lost the ability to utilize C_30_, C_32_, C_34_, and C_38_ and could only grow poorly on C_26_ and C_28_ (Fig. 1C through H). The Δ*almA*/P*almA* strain basically restored the growth ability on C_26_–C_38,_ which was similar to the wild type (WT).

**Fig 2:**
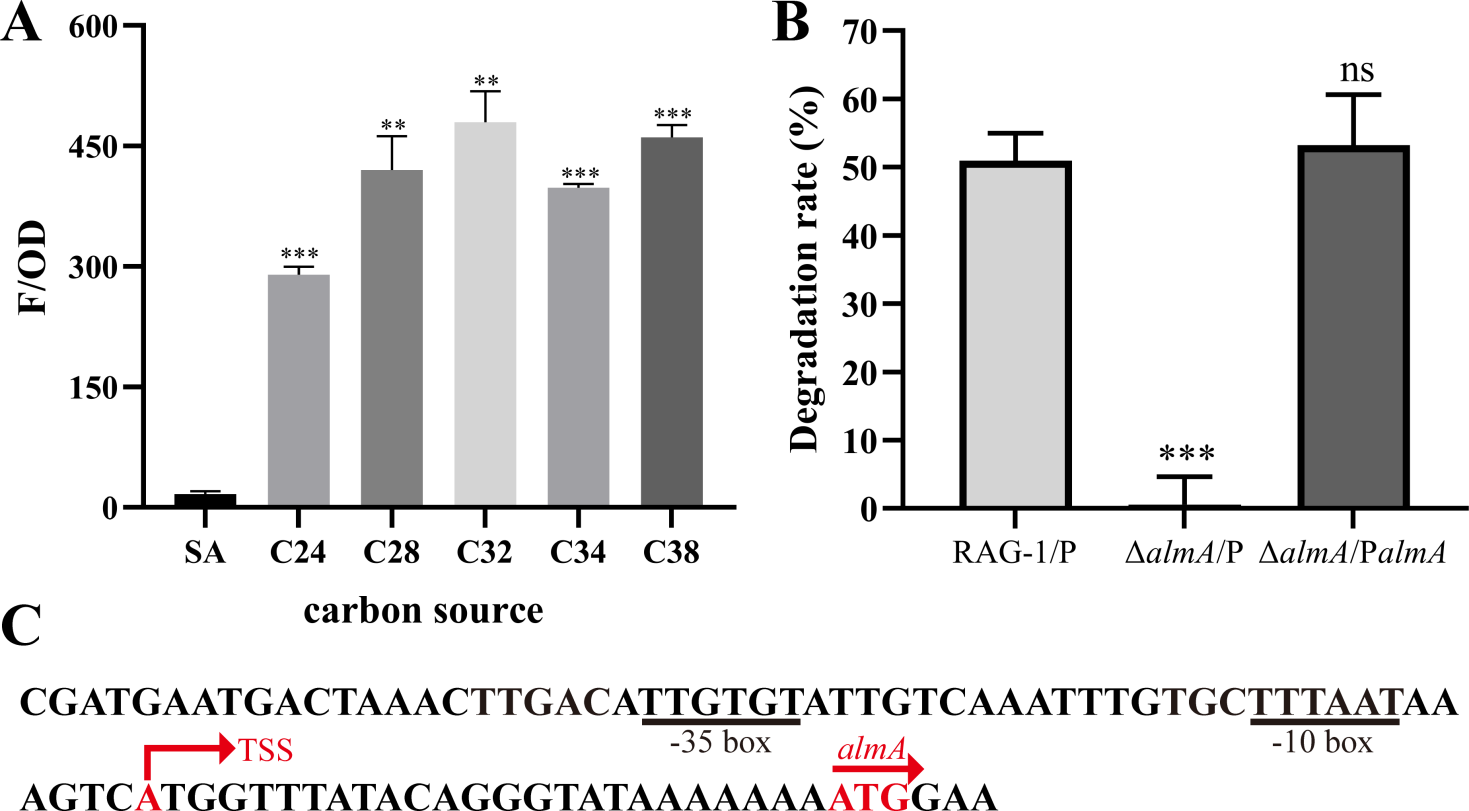
Analysis of *almA* promoter activity. (**A**) The *almA* gene promoter activity induced by SA and various long-chain *n*-alkanes was by red fluorescence expression during the logarithmic growth phase. The red fluorescence was calculated as relative fluorescence units (F/OD), which represent fluorescence values per OD_600_. (**B**) Degradation rates of RAG-1/P, △*almA*/P, and △*almA*/P*almA* strains cultured in BSM medium with *n*-C_32_ as the sole carbon source for 5 days. Values are shown as mean ± SD (*n* = 3), and the asterisk indicates a significant difference (**P < 0.01; ***P < 0.001; *ns*, not significant) by Student’s *t*-test. (**C**) DNA elements of the *almA* gene promoter. The putative −35 and the −10 regions of the *almA* gene promoter are shown underlined. The transcription start site (TSS) and start codon are shown in red.

To further investigate the expression level of *almA* under C_24_–C_38_, the vector with its promoter-*mcherry* reporter was constructed and transformed into RAG-1. When the reporter strain grown on C_24_–C_38_ was cultured to logarithmic phase, cells were used to determine the relative fluorescence value (F/OD_600_), and it was found that long-chain *n*-alkanes (C_24_–C_38_) could significantly induce the expression of *almA* in comparison with SA (Fig. 2A). The highest F/OD_600_ was detected under C_32_, suggesting that the AlmA protein was expressed at high levels and fully involved in the metabolic pathway of C_32_. Results above implied that AlmA has an essential function in the RAG-1 metabolism of C_26_–C_38_.

### APR1 as an activator of the *almA* gene

#### Analysis of the *almA* gene promoter region

To determine the transcription start site (TSS) of AlmA, the total RNA of RAG-1 cultured with C_32_ was extracted, and then 5′ RACE was performed using reverse-transcribed cDNA as the template. The rationale behind this is that the deletion of *almA* resulted in the complete inability of the strain to utilize C_32_ for growth, and the highest *almA* promoter activity is induced by C_32_ (Fig. 1F, 2A and B). As illustrated in Fig. 2C, the TSS of *almA* was identified as the “A” nucleotide located 25 bp upstream of the start codon (Fig. 2C; Fig. S2A). Based on the promoter prediction of the TSS of *almA*, its −10 box and −35 box were 5′-TTTAAT-3′ and 5′-TTGTGT-3′, respectively.

#### Identification of the transcriptional regulator of *almA*

**Fig 3:**
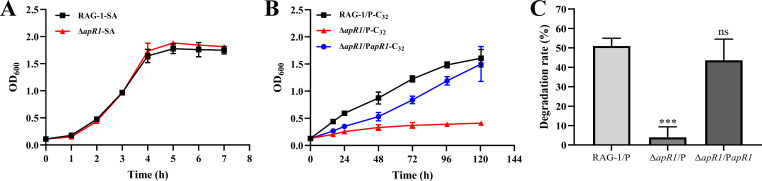
Effect of APR1 on the ability of RAG-1 to utilize *n*-C_32_. (**A**) Growth curves of RAG-1 and △*apR1* strains in BSM medium with SA as the sole carbon source; Growth curves (**B**) and degradation rates (**C**) of RAG-1/P, △*apR1*/P, and △*apR1*/P*apR1* strains cultured in BSM medium with *n*-C_32_ as the sole carbon source for 5 days. Values are shown as mean ± SD (*n* = 3), and the asterisk indicates a significant difference (***P < 0.001; *ns*, not significant) by Student’s *t*-test.

To resolve the regulatory mechanism of AlmA in RAG-1, a DNA pull-down experiment was applied to identify transcriptional regulators that could mediate *almA* expression by binding to the *almA* promoter. By analyzing the mass spectrometry results, multiple genes encoding transcriptional regulators were identified. To ascertain whether these genes are involved in C_32_ metabolism, single-gene deletion strains were constructed, and mutants were subjected to a C_32_ degradation experiment (data not shown). Only ΔF959_RS01255 mutant exhibited a notable growth deficiency on C_32_, suggesting that the F959_RS01255 gene (*apR1*) might be responsible for C_32_ metabolism. To further verify the necessity of APR1 for RAG-1 to degrade C_32_, gene deletion (Δ*apR1*/P) and complemented (Δ*apR1*/P*apR1*) strains were constructed. Growth and degradation results demonstrated that the knockout of the *apR1* markedly diminished the capacity of Δ*apR1*/P to utilize C_32_ (Fig. 3B and C), while exhibiting no impact on SA (Fig. 3A). Furthermore, the growth of the Δ*apR1*/P was also observed to be reduced on C_26_, C_28_, C_30_, C_34_, and C_38_ compared to WT. And the Δ*apR1*/P could restore the growth and degradation ability of long-chain *n*-alkanes (C_26_–C_38_) through gene complementation (Fig. S3). These results were largely consistent with the phenotype of *almA* gene deletion, thereby proving a correlation between APR1 and AlmA. Consequently, conserved domain analysis revealed that APR1 belongs to the AraC/XylS family (Fig. S4) and clusters phylogenetically with other transcriptional regulators of the AraC/XylS family that have been reported in *n*-alkane-degrading bacteria (Fig. S5).

#### Activation of the *almA* expression by APR1

**Fig 4:**
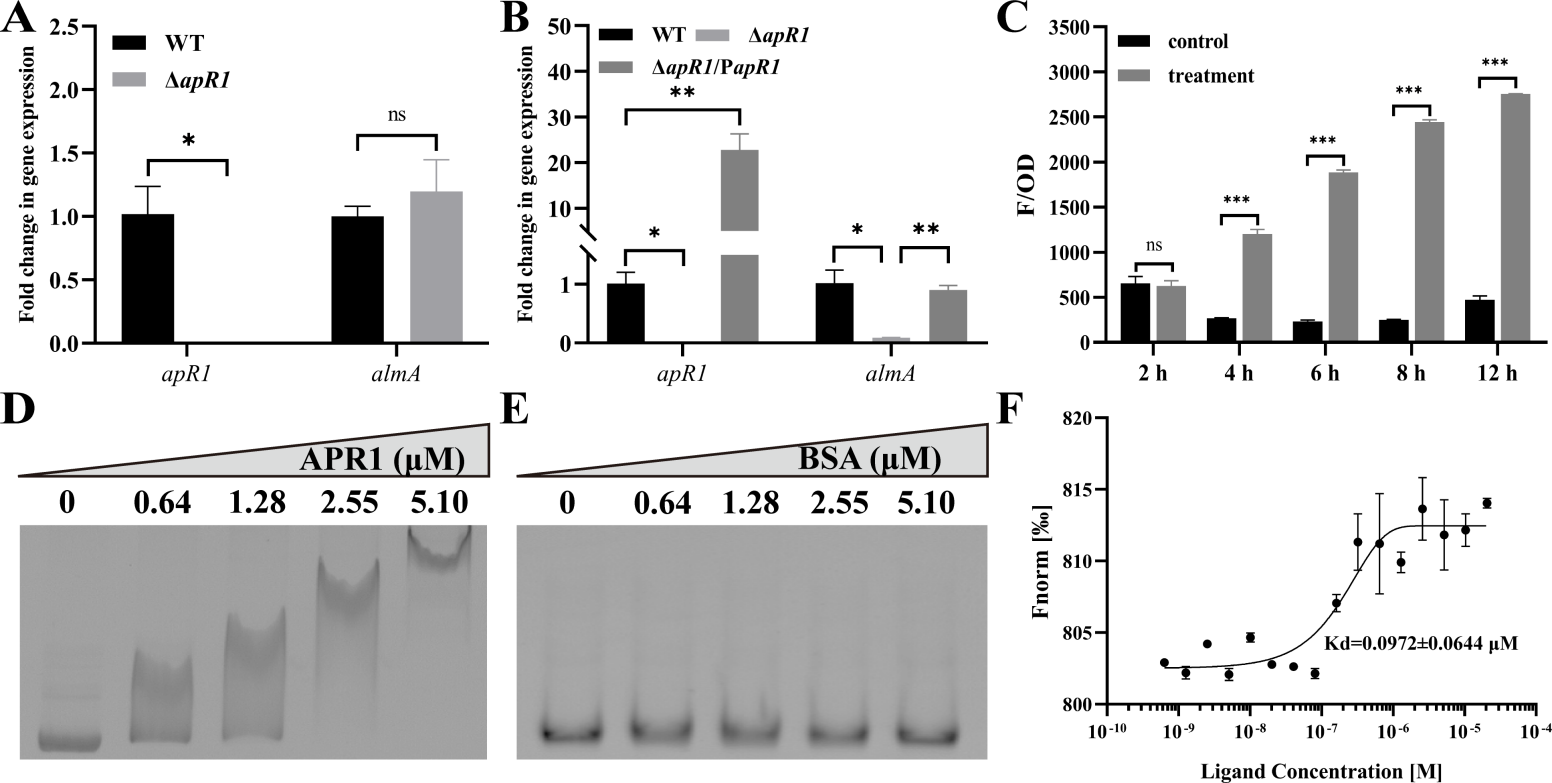
Binding of APR1 to *almA* gene promoter. (**A and B**) qRT-PCR analysis of *apR1* and *almA* gene transcription levels in WT, △*apR1*, and △*apR1*/P*apR1* strains grown in BSM medium with SA (**A**) and *n*-C_32_ (**B**) as the sole carbon source. The 16S rRNA gene was used as an internal standard gene. WT, RAG-1. (**C**) Detection of red fluorescence intensity from the co-transformed cells at different times. Reporter vectors with the *almA* gene promoter and *apR1* expression vectors were co-transformed into *E. coli* DH5α to determine red fluorescence intensity. Control, pUC18 + P*_almA_-mcherry*; treatment, pUC18-*apR1* + P*_almA_-mcherry*. The red fluorescence was calculated as relative fluorescence units (F/OD), which represent fluorescence values per OD_600_. (**D and E**) (EMSAs) on the binding of APR1 (**D**) and BSA (**E**) to *almA* gene promoter region. EMSAs with the DNA probe (110 nM) and purified APR1 and BSA (0–5.10 µM). (**F**) Binding affinities between APR1 and *almA* gene promoter region were measured by MST. NT-647-NHS labeled APR1 (10 µM) was incubated with 0.64–41,400 nM *almA* gene promoter. Values are shown as mean ± SD (*n* = 3), and the asterisk indicates a significant difference (*P < 0.05; **P < 0.01; ***P < 0.001; *ns*, not significant) by Student’s *t*-test.

For subsequent characterization, an N-terminal 6 × His-tagged APR1 was overexpressed in *Escherichia coli* BL21 (DE3) and purified by affinity chromatography. The molecular mass of 6 × His-tagged APR1 was determined to be in accordance with the theoretical value of 39.2 kDa, as indicated by SDS-PAGE (Fig. S6A). The result of glutaraldehyde cross-linking suggested that the 6 × His-tagged APR1 could dimerize (Fig. S6B), which is consistent with the reported of AraC in *E. coli*. In order to determine the relationship between APR1 and AlmA, a series of *in vivo* experiments were performed. The qPCR results demonstrated that the expression of the *almA* gene was significantly diminished when *apR1* was knocked out, and the *almA* expression could be restored after the *apR1* gene was complemented (Fig. 4B). However, this phenomenon was observed exclusively when △*apR1* was cultivated on C_32_, whereas the deletion of the *apR1* gene did not affect *almA* expression when the carbon source was SA (Fig. 4A). This suggested that APR1 might be able to activate the transcription of *almA* using C_32_ as the inducer. Subsequently, an APR1-dependent reporter system was constructed to test whether there was a direct interaction between APR1 and AlmA. pUC18 and pUC18-*apR1* were co-transformed with P_*almA*_-*mcherry* into *E. coli* DH5α and cultured in LB medium, respectively. Compared with the control group (pUC18 + P_*almA*_-*mcherry*), the F/OD_600_ of the treatment group (pUC18- *apR1* + P_*almA*_-*mcherry*) was markedly elevated (Fig. 4C), which provided preliminary evidence that APR1 directly bound the promoter region of *almA*. Then, electrophoretic mobility shift assay (EMSA) and microscale thermophoresis (MST) assays *in vitro* were carried out. EMSA was conducted by incubating various amounts of APR1 with the promoter of *almA* (110 nM), which is judged by the presence or absence of DNA probe displacement. As illustrated in Fig. 4D and E, with the increase of APR1 concentration (0–5.10 µM), APR1 bound to the *almA* promoter region, while bovine serum albumin (BSA) demonstrated no binding capacity to the same region. This result was also confirmed by the MST assay, with a *K*_d_ value of 0.0972 ± 0.0644 µM (Fig. 4F). The aforementioned findings collectively indicated that APR1 is able to positively regulate *almA* expression by directly binding to the promoter region of *almA* and that C_32_ serves as an effector that activates this response.

### Negative regulation of APR1 self-expression

**Fig 5:**
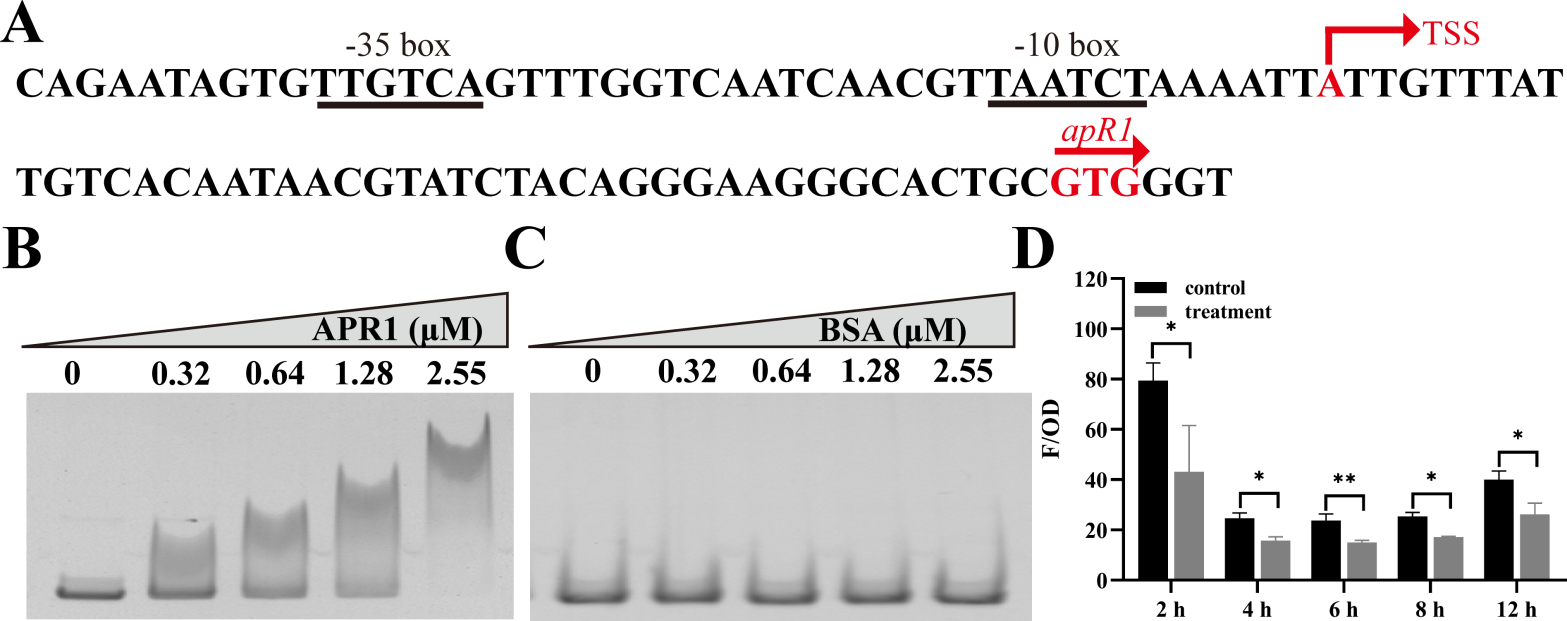
Binding of APR1 to its own gene promoter. (**A**) DNA elements of the *apR1* gene promoter. The putative −35 and the −10 regions of the *apR1* gene promoter are shown underlined. The TSS and start codon are shown in red. (**B and C**) EMSAs on the binding of APR1 (**B**) and BSA (**C**) to *apR1* gene promoter region. EMSAs with the DNA probe (140 nM) and purified APR1 and BSA (0–2.55 µM). (**D**) Detection of red fluorescence intensity from the co-transformed cells at different times. Reporter vectors with the *apR1* gene promoter and *apR1* expression vectors were co-transformed into *E. coli* DH5α to determine red fluorescence intensity. Control, pUC18 + P*_apR1_-mcherry*; treatment, pUC18-*apR1* + P*_apR1_-mcherry*. The red fluorescence was calculated as relative fluorescence units (F/OD), which represent fluorescence values per OD_600_. Values are shown as mean ± SD (*n* = 3), and an asterisk indicates a significant difference (*P < 0.05; **P < 0.01) by Student’s *t*-test.

Prior research has indicated that in *Pseudomonas oleovorans* GPo1, the transcriptional regulator AlkS can alter the level of self-expression by binding to promoter *PalkS*. Additionally, the majority of Arac/XylS family members are known to engage in self-regulation (28, 30, 31). To ascertain whether APR1 regulates its own expression at the transcriptional level, we predicted its promoter region based on its TSS (Fig. S2B). The −10 and −35 boxes were identified as 5′-TAATCT-3′ and 5′-TTGTCA-3′, respectively (Fig. 5A). EMSA results showed that APR1 was capable of directly binding to the *apR1* promoter region (140 nM) and exhibited no binding with BSA (Fig. 5B and C). The APR1-dependent reporter system in *E. coli* DH5α suggested that the relative *mcherry* expression level of the treatment group (pUC18-*apR1* + *P _apR1_-mcherry*) was notably weakened contrast to the control group (pUC18 + P*_apR1_-mcherry*) (Fig. 5D). In conclusion, it was implied that APR1 negatively regulates its own expression by directly binding to its own promoter region in the absence of exogenous long-chain *n*-alkanes.

### *n*-C_32_ functions as a ligand for APR1

In previous reports, AlkS may move to the periphery of the inner side of the cytoplasmic membrane in the presence of alkanes. This relocation allows AlkS to access alkanes and bind to the *alkB* gene promoter region, thereby activating the *alkB* expression in *P. oleovorans* GPo1 (28). However, there is currently no evidence to suggest that alkanes can induce any alterations in AlkS. Since AlmA in RAG-1 is an intracellular enzyme and that a putative ligand-binding domain is present at the N-terminus of the APR1 (Fig. S4). It is hypothesized that following the cellular uptake of long-chain *n*-alkanes via the alkane transporter, APR1 recognizes the alkane effector and binds to the *almA* promoter region through its C-terminal DNA-binding domain (Fig. S4), thereby activating the transcription of *almA*.

**Fig 6:**
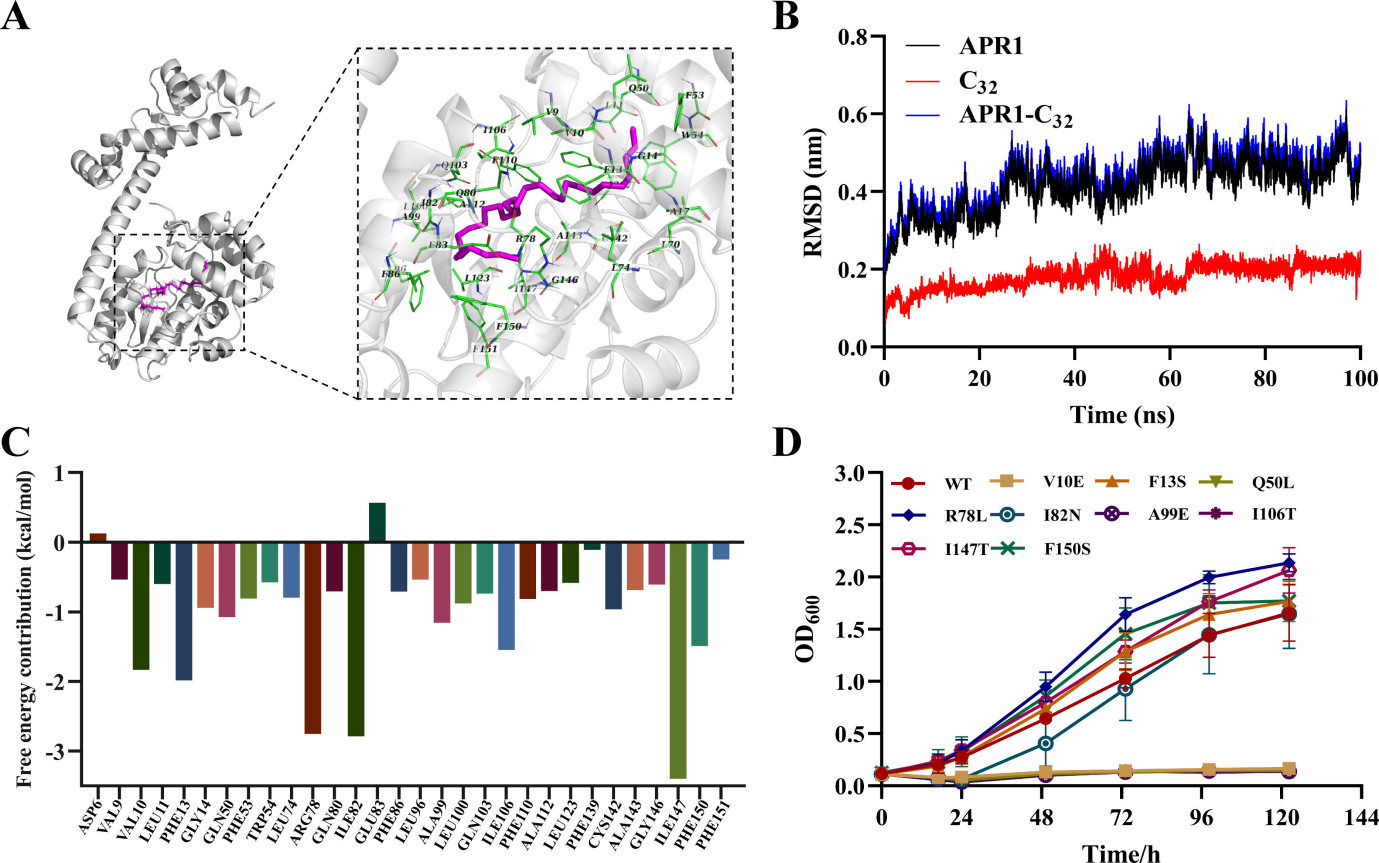
Molecular docking and dynamic simulation results of APR1-C_32_. (**A**) Docking analysis of APR1 with substrate *n*-C_32_. The *n*-C_32_ is shown in purple. (**B**) Root-mean-square deviation (RMSD) values of APR1-C_32_ complex systems over 100 ns. (**C**) Energy contribution of hot residue during simulation of APR1 and *n*-C_32_ dynamics. (**D**) Growth curves of WT and its point mutants (V10E, F13S, Q50L, R78L, I82N, A99E, I106T, I147T, F150S) cultured in BSM medium with *n*-C_32_ as the sole carbon source for 5 days. Values are shown as means ± SD (*n* = 3).

To substantiate this hypothesis, molecular docking and dynamics (MD) simulations were conducted with *n*-C_32_ using the structure by AlphaFold predicted. The MD results showed that APR1 bound to *n*-C_32_ predominantly through hydrophobic interactions, mainly due to the exceptional hydrophobicity of *n*-C_32_, and 30 key amino acid residues involved in the binding were identified (Fig. 6A). To evaluate the stability of the complex, MD simulations were employed and the root mean square deviation (RMSD) value of APR1 + *n-*C_32_ complex exhibited a slight increase during the initial phase, followed by a plateau at approximately 25 ns (Fig. 6B). The total binding force between *n*-C_32_ and APR1 was primarily determined by van der Waals energy (VDWAALS) (Table S1). By analyzing the free energy contributions of 30 amino acid residues, the amino acids with larger contributions (<−1 kcal/mol) were selected to construct mutants V10E, F13S, Q50L, R78L, I82T, A99E, I106T, I147T, and F150S via site-directed mutagenesis on genome, respectively (Fig. 6C). The growth curves evidenced that mutant strains V10E, Q50L, A99E, and I106T exhibited a substantial loss of the capacity to utilize *n*-C_32_ for growth. Conversely, the growth phenotypes of strains F13S, R78L, I147T, and F150S were stronger than those of the WT (Fig. 6D). These results indicated that Val10, Gln50, Ala99, and Ile106 amino acid residues of ligand-binding domain of APR1 are crucial for the binding of *n*-C_32_.

### Localization of the key site for APR1 binding to the *almA*/*apR1* promoter region

**Fig 7:**
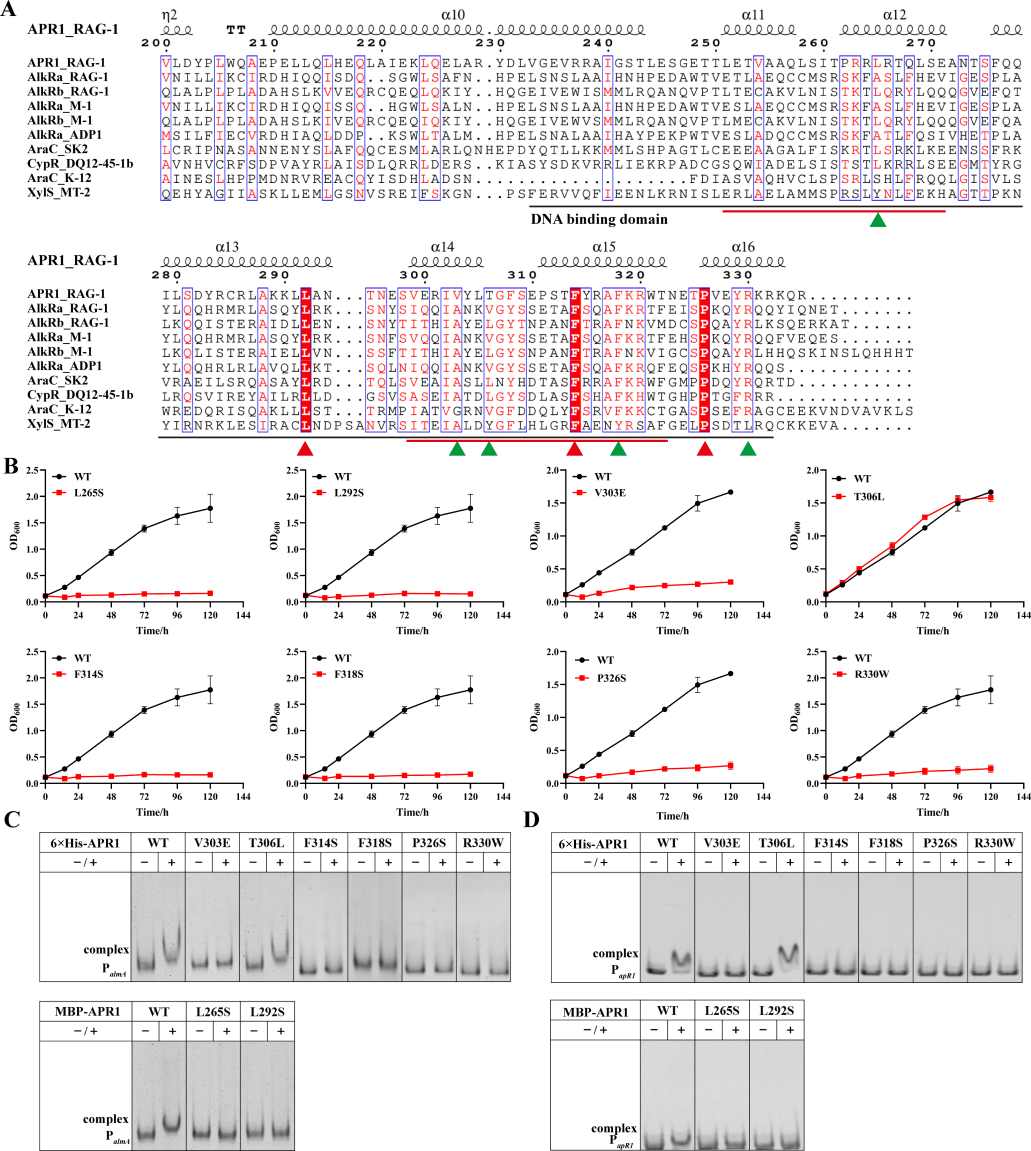
Key sites for APR1 binding to downstream promoters. (**A**) MSAs of APR1 and other Arac/XylS family transcriptional regulators of hydrocarbon-degrading bacteria. The secondary structural elements of APR1_RAG-1 are listed above the sequence. The red-filled boxes represent completely conserved sequences in these strains, and the unfilled boxes represent relatively conserved sequences. The DNA-binding domain and motifs are underlined in black and red, respectively. Red and green triangles indicate sites where point mutations were performed. (**B**) Growth curves of WT and its point mutants (L265S, L292S, V303E, T306L, F314S, F318S, F326S, R330W) cultured in BSM medium with *n*-C32 as the sole carbon source for 5 days. Values are shown as means ± SD (*n* = 3). The binding of the WT 6 × His-APR1 and its point mutants or MBP-APR1 and its point mutants to (**C**) the *almA* promoter region (110 nM) or (**D**) the *apR1* promoter region (140 nM) was measured by EMSA. −, no APR1 was added; ＋, 2.55 µM APR1 was added.

Currently, APR1 is understood to be an AraC-type transcriptional regulator that has been reported to regulate the longest-chain alkane (up to *n*-C_38_) degradation in alkane-degrading bacteria (Table 1). A previous study reported the presence of two potential helix-turn-helix (HTH) motifs at the C terminus of AraC in *E. coli*, the first of which may be responsible for recognizing different target sequences of homologous promoters. In contrast, the second may have a common function for all members (29, 30). A multiple sequence alignment (MSA) was conducted between APR1 and other alkane transcriptional regulators of the AraC/XylS family, and two HTH motifs (positions 251–271, 299–322) in APR1 were found based on the AraC of *E. coli* (Fig. 7A).

**TABLE 1:** Comparison of APR1 and other Arac/XylS family transcriptional regulators of hydrocarbon-degrading bacteria

Protein	Locus tag	Length (aa)*^a^*	Sequence identity [(*n* %)]^*b*^	Substrate(s)	Strain	Reference
APR1	WP_004876667.1	335		C_22_–C_38_*n*-alkanes	*A. venetianus* RAG-1	
AlkRa	WP_004879358.1	310	26/84 (31%)	C_16_–C_28_*n*-alkanes	*A. venetianus* RAG-1	(21)
AlkRb	WP_004880165.1	343	68/285 (24%)	C_10_–C_24_*n*-alkanes	*A. venetianus* RAG-1	(21)
CypR	AFY63002.1	333	76/311 (24%)	C_8_–C_14_*n*-alkanes	*Dietzia* sp. DQ12-45-1b	(27)
AraC	CAL15647.1	371	74/285 (26%)	Not tested	*Alcanivorax Borkumensis* SK2	(32)
AlkR	WP_004925571.1	306	24/84 (29%)	C_7_–C_18_*n*-alkanes	*Acinetobacter* sp. ADP1	(24)
AlkRa	BAB33283.1	318	26/84 (31%)	C_22_–C_30_*n*-alkanes	*Acinetobacter* sp. M1	(25)
AlkRb	BAB33288.1	317	62/285 (22%)	C_16_–C_22_*n*-alkanes	*Acinetobacter* sp. M1	(25)

^*a*^ aa, amino acids.

^*b*^ Identities were calculated using BLASTP.

Some mutant strains (Fig. 7B) and proteins (Fig. S7) were constructed by site-directed mutagenesis to further identify the key amino acid residues of APR1. The amino acid residue Leu265 of the first HTH motif is a common amino acid residue of transcriptional regulators that control the expression of alkane hydroxylases, which is only relatively conserved in the transcriptional regulators of hydrocarbon-degrading bacteria relative to AraC of *E. coli* and XylS of *P. putida* (Fig. 7A). As illustrated in Fig. 7B, the mutant strain L265S grows poorly on C_32_. Moreover, the mutant protein L265S also failed to bind to AlmA and its own promoter region (Fig. 7C and D). In conclusion, it can be proposed that Leu265 may play an important role in the recognition of the alkane hydroxylase promoter by AraC/XylS family transcriptional regulators. Subsequently, the relatively conserved (Val303, Phe318) and completely conserved (Phe315) amino acid residues of the second HTH motif were used for investigation of function through site-directed mutagenesis. The growth of mutant strains V303E, F315S, and F318S exhibited a reduction in growth on C_32_ (Fig. 7B), and none of the three mutant proteins were able to bind to the promoter (Fig. 7C and D), which served to further validate the important function of the second HTH motif. Furthermore, the role of amino acid residues in other α-helices within the DNA-binding domain was investigated, including the completely conserved Leu292 (α13) and Phe326 (α16), and the relatively conserved Thr306 (between α14 and 15) and Arg330 (α16). The results demonstrated that the L292S, P326S, and R330W proteins were severely loss-of-function, but the mutant strain T306L grew normally on C_32_ consistent with WT (Fig. 7B). Additionally, the mutant protein T306L was observed to bind to AlmA and its own promoter region (Fig. 7C and D). These observations implied that other α-helices in the DNA-binding domain of the AraC/XylS-type transcriptional regulators also play crucial roles in the regulation of downstream pathways.

### Co-evolution of APR1 and AlmA in the *Proteobacteria* class

**Fig 8:**
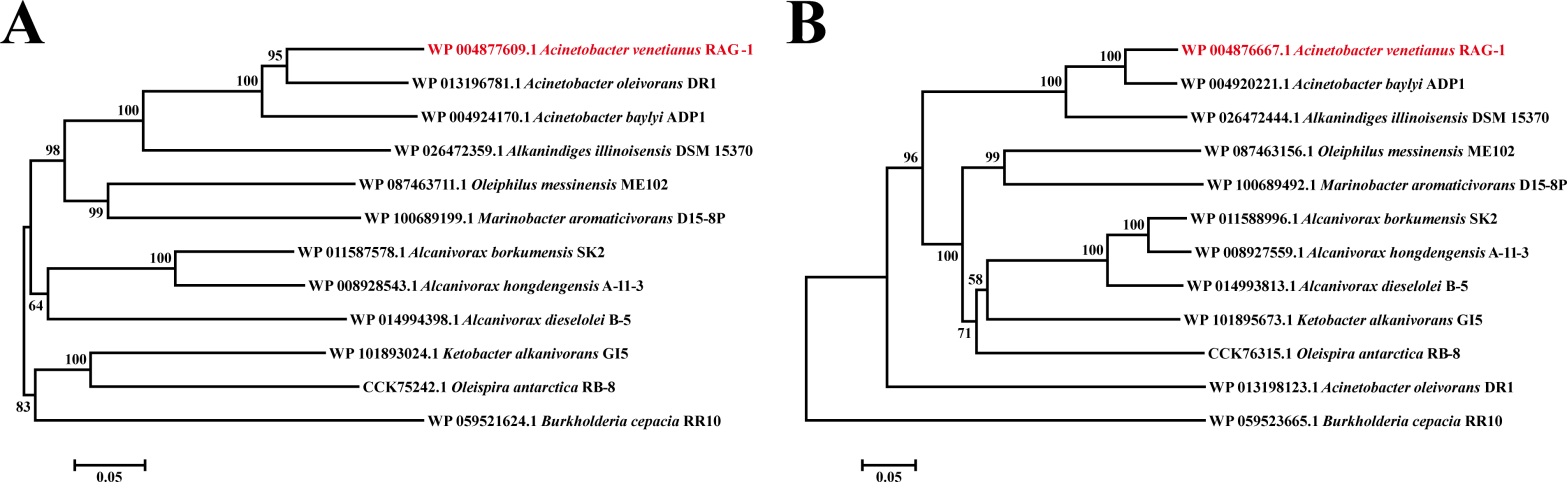
Phylogenetic analysis based on the complete amino acid sequences of AlmA and APR1. (**A**) AlmA and its homologs or (**B**) APR1 and its homologs from different strains in *Proteobacteria* were used to construct a phylogenetic tree by the neighbor-joining algorithm (1,000 replicates). The AlmA and APR1 from *A. venetianus* RAG-1 are marked with red. Bootstrap values (%) are indicated at the branch nodes, and the scale bar represents 0.05 substitutions per site.

To explore the ecophysiological status of the transcriptional regulator APR1, MSA was performed by comparing its amino acid residues with known hydrocarbon-degrading bacteria. This analysis revealed that most hydrocarbon-degrading strains containing APR1 homologs belonged to the class *Proteobacteria* (Table S2). Sequence analysis showed that APR1 exhibited 45%–90% identity with APR1 homologs in *Gammaproteobacteria* (Fig. S8). Furthermore, the putative ligand-binding domain (IPR032687) and the DNA-binding domain (IPR018060) are also relatively conserved. These results indicated that APR1 homologs might perform similar functions in these hydrocarbon-degrading strains, which possess a corresponding alkane response mechanism in them. In addition, the phylogenetic tree was constructed using APR1 homologs from the *Proteobacteria* class. APR1 was found to cluster phylogenetically with APR1 homologs of *Gammaproteobacteria*, particularly those of the *Acinetobacter* genus, and diverge from *Burkholderia cepacia* of *Betaproteobacteria* (Fig. 8B). The evolutionary trajectory of AlmA was basically consistent with that of APR1, i.e., the amino acid sequence of AlmA clustered phylogenetically with those of AlmA homologs that belong to the *Gammaproteobacteria* (Fig. 8A). In summary, these indicated that APR1 and AlmA have a similar phylogenetic process in the *Proteobacteria* class. However, the organization and composition of the *almA* and *apR1* gene clusters are diverse. They are variable across genus but have certain similarity between the same genus, e.g., *Acinetobacter* spp. and *Alcanivorax* spp. (Fig. S9 and S10 ).

## DISCUSSION

**Fig 9:**
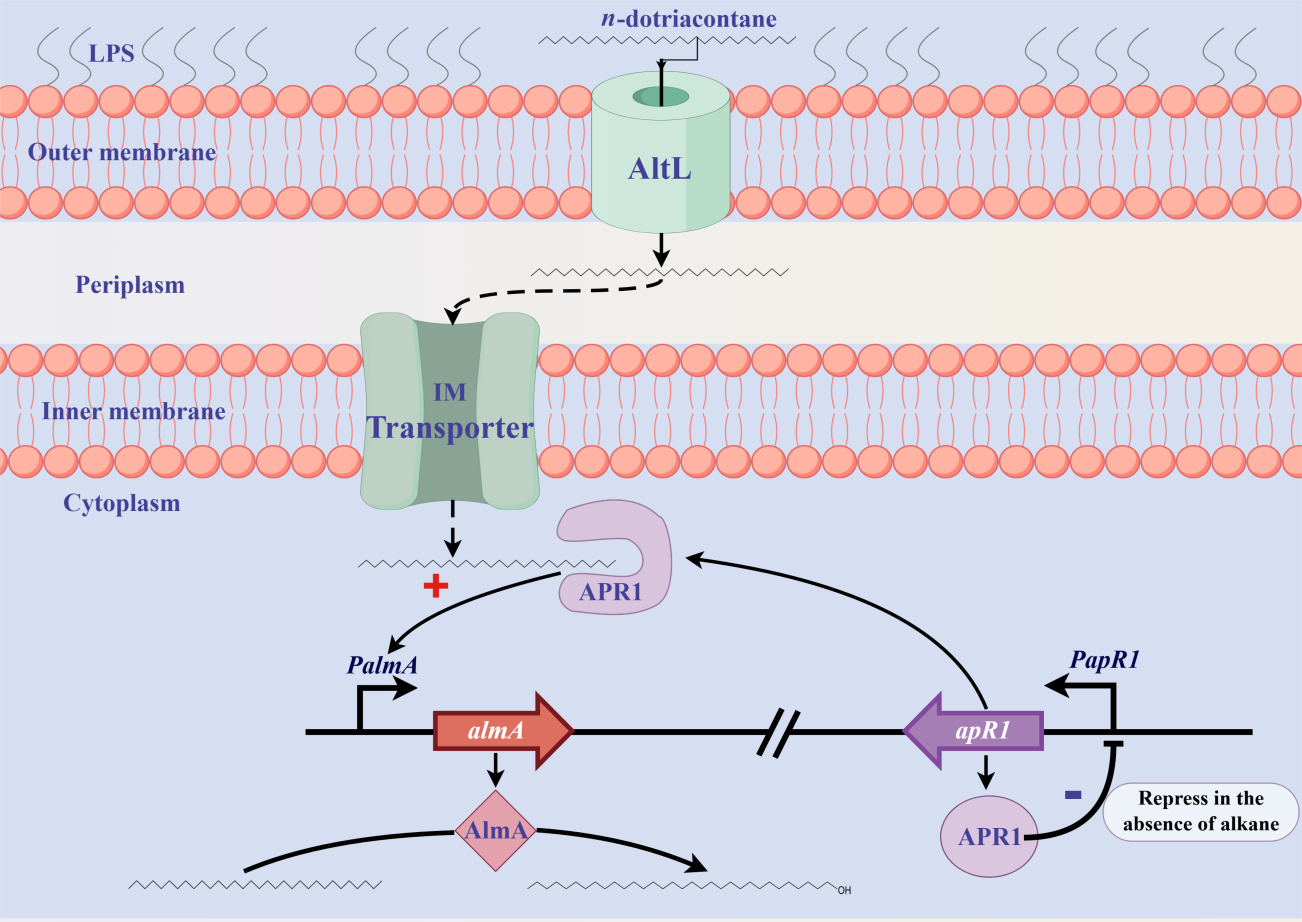
The proposed regulatory mechanism of AlmA in RAG-1 cells. Please refer to the initial paragraph of the Discussion section for further details.

In this study, an AraC/XylS family transcriptional activator APR1 was identified. The *apR1* gene deletion mutant exhibited a markedly diminished capacity to degrade C_26_–C_38_ *n*-alkanes, and the phenotype was restored by gene complementation (Fig. S3). APR1-dependent reporter system, qRT-PCR, EMSA, and MST experiments showed that APR1 was able to bind directly to the promoter of *almA* and activate its expression using *n*-C_32_ as the inducer (Fig. 4). Furthermore, molecular docking, dynamics simulation, and site-directed mutagenesis of key amino acid residues revealed that *n*-C_32_ could function as a ligand for APR1 (Fig. 6). Therefore, combining on our above findings and previous work, we proposed a model (Fig. 9). The outer membrane protein AltL is responsible for the transport of *n*-C_32_ to the periplasm. Following this, the unidentified inner membrane transporter is likely be involved in the entry of *n*-C_32_ into the cytoplasm. Subsequently, the regulator APR1 recognizes the *n*-C_32_ and binds to the *almA* promoter region, thereby activating the expression of *almA*, which is involved in the *n*-C_32_ metabolic pathway. Meanwhile, in the absence of *n*-C_32_, APR1 represses its own expression. Nevertheless, our current research has only focused on the mechanism of APR1 in response to *n*-C_32_, and we have not yet investigated the effects of other long-chain *n*-alkanes. Based on the growth of Δ*apR1* and Δ*almA* mutants under alkanes, it can be tentatively assumed that APR1 also responds by effectors C_26_–C_38_, a hypothesis that warrants further explored.

In our previous study, transcriptomic analysis revealed that three alkane hydroxylase genes (*alkMa*, *alkMb,* and *almA*) exhibited a notable increase in expression when *A. venetianus* RAG-1 was cultured on long-chain *n*-alkanes. The growth and degradation of single- and multi-deletion mutants on *n*-alkanes confirmed that AlkMa was responsible for C_16_–C_28_ oxidation, AlkMb for C_10_–C_24_ oxidation, and AlmA for C_22_–C_38_ oxidation (21). The functional redundancy of the three hydroxylases may facilitate the utilization of a broader range of *n*-alkanes by RAG-1, thereby enabling adaptation to a complex external environment. This type of coexistence of multiple hydroxylase systems has been observed in other strains. In *P. aeruginosa* SJTD-1, two AlkB-type proteins (AlkB1 and AlkB2), two P450 monooxygenases, and an AlmA-type monooxygenase have been identified and characterized as being involved in the C_12_–C_24_ degradation pathway (33, 34). In *Acinetobacter* sp. strain DSM17874, two *alkB*-type genes (*alkMa* and *alkMb*) and an *almA* gene were found, with AlkMa and AlkMb showing the ability to oxidize C_10_–C_20_ and AlmA affecting the utilization of C_32_–C_36_ (16, 35). In this study, the growth of the single-deletion mutant Δ*almA* on long-chain *n*-alkanes was analyzed, demonstrating that AlmA was involved in the C_26_–C_38_ degradation pathway, but the impact on the degradation of C_22_–C_24_ could not be verified due to gene redundancy. However, the growth of Δ*apR1* was significantly reduced on C_22_–C_38_, and the result was consistent with the previously reported range of substrate utilization by AlmA. This further validates the positive correlation between APR1 and AlmA. The Δ*almA* mutant exhibited a completely lost of the ability to use C_30_–C_38_ for growth, whereas the Δ*apR1* mutant demonstrated the retention of this capacity. These results implied the possibility of additional activators of *almA* in RAG-1. This conclusion was further corroborated by qPCR experiments, which yielded evidence of residual expression of *almA* following the knockdown of *apR1*. A similar phenomenon is also observed in *Pseudomonas aeruginosa*, whereby a single gene is regulated by multiple regulators. Both the AraC/XylS-type transcription factor VqsM and the global regulator Vfr have been identified as activators of *exsA* by directly binding to its promoter region (36, 37). Conversely, MvaT and MvaU have been shown to repress the expression of the TSS genes by binding to *exsA* promoter region (38). These findings collectively illustrate the intricate regulatory mechanisms governing the metabolism of long-chain *n*-alkanes in hydrocarbon-degrading strains.

Currently, many reported alkane transcriptional regulators are located on the genome adjacent to their target genes. To illustrate, in *Acinetobacter* sp. strain ADP1, the alkane monooxygenase AlkM can be activated by its upstream transcriptional regulator AlkR, whose presence is required for AlkM expression (24). Moreover, AlmR, the sole-reported repressor of *almA* in *A. dieselolei* B5, is positioned in close proximity to the upstream region of *almA* (19). However, the location of *apR1* was not adjacent to *almA* on the RAG-1 genome. According to the prediction, there is an Ig-like domain in AlmR (Fig. S11), unlike APR1. Meanwhile, the amino acid sequences of APR1 and AlmR exhibited only 4.86% identity (Table S3). The phylogenetic tree analysis for the homologs of APR1 and AlmR demonstrated that they were distinctly partitioned into two branches (Fig. S12). The above results provide further evidence that they may have disparate functions. Furthermore, a search for genes near *almA* revealed the presence of the gene F959_RS03490, which exhibited a transcription orientation opposite to that of *almA* transcription. Through conserved domain analysis, this gene was identified as lacking an HTH motif. In conclusion, our findings highlight a divergence from the previously proposed proximity regulation model of alkane degradation with respect to the regulation of *almA* in RAG-1. This observation broadens the existing knowledge base on the regulation of hydrocarbon degradation and provides novel insights that could inform future research on the regulatory mechanisms governing this process.

The majority of members of the AraC/XylS family are self-regulatory (30). It has been observed that the AraC of *E. coli* inhibits self-expression by binding to the RNA polymerase recognition site of its own promoter (31). In the *n*-alkane metabolic pathway of *Pseudomonas fluorescens* GPo1, AlkS activates the transcription of the *alkBFGHJKL* operon, and also AlkS negatively regulates the expression of the *alkST* gene by binding to its own promoter to keep its own expression at a low level in the absence of exogenous alkanes (28). Conversely, the presence of alkanes enables AlkS to positively regulate its own expression, thereby activating the *alkBFGHJKL* operon and facilitating the degradation of *n*-alkanes. Similarly, APR1 is also capable of self-regulation, and when induced by the absence of alkanes, APR1 can inhibit its own expression.

*A. venetianus* RAG-1 is a Gram-negative bacterium, and its cell membrane is subdivided into an inner and an outer membrane (39, 40). Some studies have observed that *n*-alkanes (C_1_–C_8_) may traverse the cell membrane via diffusion, while long-chain *n*-alkanes are more hydrophobic and may accumulate within the cell membrane (41). In a previous study, we characterized an outer membrane transporter protein AltL in RAG-1, which was responsible for the transport of long-chain *n*-alkanes (C_20_–C_38_) and fatty acids (C_18A_–C_28A_) (2). AlmA is a soluble intracellular enzyme that, unlike the membrane-integrated AlkB (42), is unable to oxidize *n*-alkanes directly in the cell membrane. Moreover, the N-terminus of APR1 has been identified as a potential ligand-binding domain. Consequently, it was hypothesized that long-chain *n*-alkanes would enter the cytosol via alkane transporters on the inner membrane and act as ligands for APR1. The MD revealed that complex retained its structural integrity following the binding of APR1 to *n*-C_32_, while key amino acid residues Val10, Gln50, Ala99, and Ile106 were identified, thereby corroborating the possibility of this hypothesis. This differs from the reported regulatory mechanism of *alkB* in *P. fluorescens* GPo1 and *almA* in *A. dieselolei* B5. In *Pseudomonas fluorescens* GPo1, the expression of *alkB* is dependent on the transcriptional activator AlkS (28). It is possible that AlkS has an affinity for the inner membrane and, thus, recognized *n*-alkanes to initiate the *alkBFGHJKL* operon transcription. The *almR* gene is initially regulated by the global transcriptional regulator CoyD protein, which then represses *almA* expression in *A. dieselolei* B5 (19).

A common feature of bacterial transcriptional regulators is the capacity to bind target DNA through HTH motifs (43). Among them, the motif positions in AraC of *E. coli* are 195 to 218 and 245 to 270 (30). The HTH motifs of APR1 were identified via MSA and domain prediction, with the positions being 251 to 271 and 299 to 322. In the first motif, *n*-alkane transcriptional regulators display a conserved amino acid residue (Phe265) that differs from that observed in other AraC/XylS-type transcriptional regulators. The mutation of this position dramatically impaired the ability of the strain to grow on *n*-C_32_. This implies that this amino acid residue may serve a crucial role in discerning the promoter region of alkane hydroxylase gene.

In summary, this study focused on the identification of APR1, an activator of the *almA* gene, which was mainly responsible for activating the transcription of *almA* by binding to its promoter region upon recognition of the ligand long-chain *n*-alkanes. Our study has extended the known members of the AraC/XylS-type transcriptional regulators in the alkane degradation pathway and has further elucidated the regulatory mechanisms governing the degradation of more hydrophobic long-chain *n*-alkanes in hydrocarbon-degrading bacteria. This is crucial for practical applications in oil spill remediation and enhanced oil recovery.

## MATERIALS AND METHODS

### Strains, plasmids, primers, media, and culture conditions

The strains, plasmids, and primers used in this study are listed in Tables 2 and 3. *E. coli* strains DH5α, S17-1, and BL21 (DE3) were cultured at 37℃ in lysogeny broth medium (LB, 10% tryptone, 5% yeast extract, 10% NaCl, pH 7.2). *A. venetianus* RAG-1 was gifted by Professor D L Gutnick (Tel Aviv University, Israel). It was grown at 30℃ in BSM [3.815 g/L K_2_HPO_4_, 0.5 g/L KH_2_PO_4_, 0.825 g/L (NH_4_)_2_HPO_4_, 1.2625 g/L KNO_3_, 0.2 g/L Na_2_SO_4_, 0.02 g/L MgCl_2_, 0.02 g/L CaCl_2_, 0.002 g/L FeCl_3_, pH 7.2] (33) supplemented with 0.2% sodium acetate (SA) or 0.1% long-chain *n*-alkanes (C_22_–C_38_). If necessary, different concentrations of antibodies are added. Kanamycin (Km), chloramphenicol (Chl), and ampicillin (Amp) were used at 50 µg/mL, 7.5 µg/mL, and 100 µg/mL, respectively.

**TABLE 2:** Strains and plasmids used in this study

Strains/plasmids	Characteristics	Source
*A. venetianus*		
RAG-1	Wild-type; *n*-alkane degrading strain	Presented by D L Gutnick
RAG-1/P	RAG-1 containing pMK plasmid	This study
RAG-1/P*_almA_-mcherry*	RAG-1 containing P*_almA_-mcherry* plasmid	This study
Δ*almA*/P	Δ*almA* containing pMK plasmid	This study
Δ*almA*/P*almA*	*almA* complemented strain	This study
Δ*apR1*	*apR1* deletion mutant	This study
Δ*apR1*/P	Δ*apR1* containing pMK plasmid	This study
Δ*apR1*/P*apR1*	*apR1* complemented strain	This study
L265S	△*apR1* with *apR1*^L265S^ *in situ* insertion on the genome	This study
L292S	△*apR1* with *apR1*^L292S^ *in situ* insertion on the genome	This study
V303E	△*apR1* with *apR1*^V303E^ *in situ* insertion on the genome	This study
T306L	△*apR1* with *apR1*^T306L^ *in situ* insertion on the genome	This study
F314S	△*apR1* with *apR1*^F314S^ *in situ* insertion on the genome	This study
F318S	△*apR1* with *apR1*^F318S^ *in situ* insertion on the genome	This study
P326S	△*apR1* with *apR1*^P326S^ *in situ* insertion on the genome	This study
R330W	△*apR1* with *apR1*^R330W^ *in situ* insertion on the genome	This study
V10E	△*apR1* with *apR1*^V10E^ *in situ* insertion on the genome	This study
F13S	△*apR1* with *apR1*^F13S^ *in situ* insertion on the genome	This study
Q50L	△*apR1* with *apR1*^Q50L^ *in situ* insertion on the genome	This study
R78L	△*apR1* with *apR1*^R78L^ *in situ* insertion on the genome	This study
I82T	△*apR1* with *apR1*^I82T^ *in situ* insertion on the genome	This study
A99E	△*apR1* with *apR1*^A99E^ *in situ* insertion on the genome	This study
I106T	△*apR1* with *apR1*^I106T^ *in situ* insertion on the genome	This study
I147T	△*apR1* with *apR1*^I147T^ *in situ* insertion on the genome	This study
F150S	△*apR1* with *apR1*^F150S^ *in situ* insertion on the genome	This study
*E. coli*		
DH5α	Cloning strain	Lab collection
S17-1	*recA* pro *hsdR* RP4-2-Tc::Mu-Km::Tn7	Lab collection
BL21 (DE3)	Expression strain	Sangon Biotech
Plasmids		
pMD19T	TA cloning vector, Amp^r^	Takara
pKU	Suicide plasmid for gene knockout, Kan^r^	Lab collection
pKU-*apR1*-UD	pKU containing the upstream and downstream homologous fragment of *apR1*, Kan^r^	This study
pKU-*apR1*^L265S^	pKU containing the upstream and downstream homologous fragment of *apR1* with *apR1*^L265S^, Kan^r^	This study
pKU-*apR1*^L292S^	pKU containing the upstream and downstream homologous fragment of *apR1* with *apR1*^L292S^, Kan^r^	This study
pKU-*apR1*^V303E^	pKU containing the upstream and downstream homologous fragment of *apR1* with *apR1*^V303E^, Kan^r^	This study
pKU-*apR1*^T306L^	pKU containing the upstream and downstream homologous fragment of *apR1* with *apR1*^T306L^, Kan^r^	This study
pKU-*apR1*^F314S^	pKU containing the upstream and downstream homologous fragment of *apR1* with *apR1*^F314S^, Kan^r^	This study
pKU-*apR1*^F318S^	pKU containing the upstream and downstream homologous fragment of *apR1* with *apR1*^F318S^, Kan^r^	This study
pKU-*apR1*^P326S^	pKU containing the upstream and downstream homologous fragment of *apR1* with *apR1*^P326S^, Kan^r^	This study
pKU-*apR1*^R330W^	pKU containing the upstream and downstream homologous fragment of *apR1* with *apR1*^R330W^, Kan^r^	This study
pKU-*apR1*^V10E^	pKU containing the upstream and downstream homologous fragment of *apR1* with *apR1*^V10E^, Kan^r^	This study
pKU-*apR1*^F13S^	pKU containing the upstream and downstream homologous fragment of *apR1* with *apR1*^F13S^, Kan^r^	This study
pKU-*apR1*^Q50L^	pKU containing the upstream and downstream homologous fragment of *apR1* with *apR1*^Q50L^, Kan^r^	This study
pKU-*apR1*^R78L^	pKU containing the upstream and downstream homologous fragment of *apR1* with *apR1*^R78L^, Kan^r^	This study
pKU-*apR1*^I82T^	pKU containing the upstream and downstream homologous fragment of *apR1* with *apR1*^I82T^, Kan^r^	This study
pKU-*apR1*^A99E^	pKU containing the upstream and downstream homologous fragment of *apR1* with *apR1*^A99E^, Kan^r^	This study
pKU-*apR1*^I106T^	pKU containing the upstream and downstream homologous fragment of *apR1* with *apR1*^I106T^, Kan^r^	This study
pKU-*apR1*^I147T^	pKU containing the upstream and downstream homologous fragment of *apR1* with *apR1*^I147T^, Kan^r^	This study
pKU-*apR1*^F150S^	pKU containing the upstream and downstream homologous fragment of *apR1* with *apR1*^F150S^, Kan^r^	This study
pMK	Expression vector, Kan^r^	(2)
P*almA*	pMK carrying the *almA* ORF, Kan^r^	This study
P*apR1*	pMK carrying the *apR1* ORF, Kan^r^	This study
pUC18	Cloning vector, Amp^r^	Lab collection
pUC18-*apR1*	pUC18 carrying the *apR1* ORF, Amp^r^	This study
P*_almA_-mcherry*	pMK containing the promoter region of *almA* with *mcherry*	This study
P*_apR1_-mcherry*	pMK containing the promoter region of *apR1* with *mcherry*	This study
pET-28a (+)	Expression vector, Kan^r^	Lab collection
pET-MBP	Expression vector, Kan^r^	Lab collection
pET-28a (+)-*apR1*	pET-28a (+) harboring *apR1* ORF, Km^r^	This study
pET-MBP-*apR1*	pET-MBP harboring *apR1* ORF, Km^r^	This study
pET-MBP-*apR1*^L265S^	pET-MBP harboring *apR1*^L265S^ ORF, Km^r^	This study
pET-MBP-*apR1*^L292S^	pET-MBP harboring *apR1*^L292S^ ORF, Km^r^	This study
pET-28a (+)-*apR1*^V303E^	pET-28a (+) harboring *apR1*^V303E^ ORF, Km^r^	This study
pET-28a (+)-*apR1*^T306L^	pET-28a (+) harboring *apR1*^T306L^ ORF, Km^r^	This study
pET-28a (+)-*apR1*^F314S^	pET-28a (+) harboring *apR1*^F314S^ ORF, Km^r^	This study
pET-28a (+)-*apR1*^F318S^	pET-28a (+) harboring *apR1*^F318S^ ORF, Km^r^	This study
pET-28a (+)-*apR1*^P326S^	pET-28a (+) harboring *apR1*^P326S^ ORF, Km^r^	This study
pET-28a (+)-*apR1*^R330W^	pET-28a (+) harboring *apR1*^R330W^ ORF, Km^r^	This study

**TABLE 3:** Primers used in this study^*a*^

Primer name	Sequence (5′−3′)	Function
16S-RTF	ACAGAGGGTGCGAGCGTTAATC	qRT-PCR
16S-RTR	CTGCCTTCGCCATCGGTATTCC	qRT-PCR
*almA*-RTF	AGGCGTTATGGTGAGCGATGTG	qRT-PCR
*almA*-RTR	GAAGGATCGGCGTGTGCAATCA	qRT-PCR
*apR1*-RTF	TTAACAGATGCATCGGTTGTATTG	qRT-PCR
*apR1*-RTR	GATATCTGGGTCATTGCTCACTTC	qRT-PCR
*apR1*-F1	GCCAAGCTTGCATGCCTGCATTATCAGCAGTTACGCAAACATCTC	gene knock-out
*apR1*-R1	CTTTAAAATTCCTCACACCAGCAGTGCCCTTCCCTGTAGA	gene knock-out
*apR1*-F2	TCTACAGGGAAGGGCACTGCTGGTGTGAGGAATTTTAAAGCC	gene knock-out
*apR1*-R2	GCATGACTTGACGGATATGCAATGAACCGATTATTGTTCATGC	gene knock-out
*apR1*^L265S^-R1	CTGTGTTCTTGAACGGCGTG	point mutation
*apR1*^L265S^-F2	CACGCCGTTCAAGAACACAG	point mutation
*apR1*^L292S^-R1	GGTATTTGCCGATAACTTTTTCG	point mutation
*apR1*^L292S^-F2	CGAAAAAGTTATCGGCAAATACC	point mutation
*apR1*^V303E^-R1	CCGTTAAATACTCAATACGCTCTAC	point mutation
*apR1*^V303E^-F2	GTAGAGCGTATTGAGTATTTAACGG	point mutation
*apR1*^T306L^-R1	TCAGAGAAACCCAATAAATACACA	point mutation
*apR1*^T306L^-F2	TGTGTATTTATTGGGTTTCTCTGA	point mutation
*apR1*^F314S^-R1	ATGCTCGATAGGACGTACTTGG	point mutation
*apR1*^F314S^-F2	CCAAGTACGTCCTATCGAGCAT	point mutation
*apR1*^F318S^-R1	CCAACGTTTAGATGCTCGATAG	point mutation
*apR1*^F318S^-F2	CTATCGAGCATCTAAACGTTGG	point mutation
*apR1*^P326S^-R2	TAAAACGACGGCCAGTGCCATTAACGTTGTTTACGCTTCCGATACTCAACTGATGTTTCA	point mutation
*apR1*^R330W^-R2	TAAAACGACGGCCAGTGCCATTAACGTTGTTTACGCTTCCAATACT	point mutation
*apR1*^V10E^-R1	CAAAGCGCAATTCAACCGAT	point mutation
*apR1*^V10E^-F2	ATCGGTTGAATTGCGCTTTG	point mutation
*apR1*^F13S^-R1	CCTGATAACCAGAGCGCAATAC	point mutation
*apR1*^F13S^-F2	GTATTGCGCTCTGGTTATCAGG	point mutation
*apR1*^Q50L^-R1	AGAATGCATTTAGTGCACTCAG	point mutation
*apR1*^Q50L^-F2	CTGAGTGCACTAAATGCATTCT	point mutation
*apR1*^R78L^-R1	CACTTGACCAAGATACAGTGGT	point mutation
*apR1*^R78L^-F2	ACCACTGTATCTTGGTCAAGTG	point mutation
*apR1*^I82T^-R1	CAAATGCTCGGTCACTTGACC	point mutation
*apR1*^I82T^-F2	GGTCAAGTGACCGAGCATTTG	point mutation
*apR1*^A99E^-R1	GATAAGCCAATTCTCGCTTGAG	point mutation
*apR1*^A99E^-F2	CTCAAGCGAGAATTGGCTTATC	point mutation
*apR1*^I106T^-R1	CAAAAGCATCACTAGTTAAACGCTGAT	point mutation
*apR1*^I106T^-F2	ATCAGCGTTTAACTAGTGATGCTTTTG	point mutation
*apR1*^I147T^-R1	AAAACGTAAGGTTCCTGACATCGC	point mutation
*apR1*^I147T^-F2	GCGATGTCAGGAACCTTACGTTTT	point mutation
*apR1*^F150S^-R1	GTAATAAATTTAAAAGAACGTAAGATTCCTG	point mutation
*apR1*^F150S^-F2	CAGGAATCTTACGTTCTTTTAAATTTATTAC	point mutation
*almA*-F	CAGGAAACAGAATTCGAGCTATGGAAAAGCAAGTTGATGTATTGA	gene complementation
*almA*-R	TCTAGAGGATCCCCGGGTACTTATGATACTAATTTTGGCTTACGGTTTG	gene complementation
*apR1*-F	CTACAGGGAAGGGCACTGCATGGGTCAGTTAACAGATGCATCG	gene complementation
*apR1*-R	CAGGAAACAGAATTCGAGCTCTACAGGGAAGGGCACTGC	gene complementation
UC-*apR1*-F	CAGCTATGACCATGATTACGATGGGTCAGTTAACAGATGCATCG	promoter activity
UC-*apR1*-R	TAAAACGACGGCCAGTGCCATTAACGTTGTTTACGCTTCCG	promoter activity
*almA-*Pro-F	CCATGAAAAATACCATGCTCTTGTTTATACTCTATTAACAGC	promoter activity
*almA-*Pro-R	TCCTCGCCCTTGCTCACCATTTTTTTTATACCCTGTATAAACCATG	promoter activity
*mcherry*-F	ATGGTGAGCAAGGGCGAGGA	promoter activity
*mcherry*-R	TCTAGAGGATCCCCGGGTACTTAGCCGGCCTTGTACAGCTC	promoter activity
*apR1-*Pro-F	CCATGAAAAATACCATGCTCAGCCTACCTGATGATCAAATTGG	promoter activity
*apR1-*Pro-R	TCCTCGCCCTTGCTCACCATGCAGTGCCCTTCCCTGTAGA	promoter activity
His-*apR1-*F	CTGGTGCCGCGCGGCAGCCATATGGGTCAGTTAACAGATGCATCG	protein purification
His-*apR1-*R	AGTGGTGGTGGTGGTGGTGCTTAACGTTGTTTACGCTTCCG	protein purification
MBP-*apR1*-F	GTATTTTCAGGGAGGATCCGAAATGGGTCAGTTAACAGATGCATCG	protein purification
MBP-*apR1-*R	AGTGGTGGTGGTGGTGGTGCTTAACGTTGTTTACGCTTCCG	protein purification
*almA*-Race-1	GCCTTTGATCTCAGCACCGCTAGCCAACACTTTGTCTTTTGCCC	5´-RACE
*almA*-Race-2	CTGAACGAATACCAGGATAGCGG	5´-RACE
*apR1*-Race-1	AACGTAAGATTCCTGACATCGCACATTCAGAAAAATGGCGATTCAC	5´-RACE
*apR1*-Race-2	CATCTTCAATCACCAACTTAGCGTC	5´-RACE
P*_almA_*-biotin-F	AATCAATTGTTTATACTCTATTAACAGC	DNA-pull down
P*_almA_*-F	AATCAATTGTTTATACTCTATTAACAGC	EMSA
P*_almA_*-R	ATACCCTGTATAAACCATGACTTT	EMSA
P*_apR1_*-F	AGCCTACCTGATGATCAAATTGG	EMSA
P*_apR1_*-R	GCAGTGCCCTTCCCTGTAGA	EMSA

^*a*^ Single and double underlined region in the primers represent mutant sites and overlap sequences, respectively.

### Genetic manipulations

The Δ*apR1* strain was generated via homologous recombination as described previously (21). The upstream and downstream flanking regions of the *apR1* gene were amplified with primer pairs *apR1*-F1/*apR1*-R1 and *apR1*-F2/*apR1*-R2, respectively. The two fragments were cloned into pKU by Gibson assembly to obtain pKU-*apR1*-UD. Then, pKU-*apR1*-UD was transformed into the S17-1 strain. Single/double-crossover mutants were screened after the crossing-over experiment of S17-1 and RAG-1. Finally, the transformants were verified by PCR amplification and sequencing. For gene complementation, the *apR1* gene was amplified using primers *apR1*-F/*apR1*-R and inserted into pMK to generate pMK-*apR1*. The recombinant vector was transformed into △*apR1* by electroporation to obtain the △*apR1*/P*apR1* strain. Furthermore, the △*almA*/P*almA* strain was obtained using the same approach.

For the acquisition of point mutations, the method used was similar to that used for *apR1* deletion. The differences are as follows. Mutation sites were designed on primers for the amplification of fragments. S17-1 strain transformed with pKU-*apR1* and △*apR1* was applied for the crossing-over experiment.

### Measurement of promoter activity

The promoter region of *almA* was amplified with primer pair *almA*-pro-F/*almA*-pro-R and then cloned into pMK with *mcherry* gene to generate P*_almA_-mcherry*. The vector was transformed into RAG-1 by electroporation. When cells cultured in BSM supplement with 0.2% SA or 0.1% long-chain *n*-alkanes grown to the logarithmic stage, samples were used for OD_600_ and fluorescence (587/610 nm) measurement on an Automated Imaging Microplate Reader, BioTek Cytation 5 (Agilent, Santa Clara, CA, United States). Fluorescence was calculated as relative fluorescence units (F/OD), which represent fluorescence values per OD_600_.

### Growth and degradation assay

The deletion, complementation, and point-mutant strains were precultured in LB to OD_600_≈2.0 and then collected by centrifugation and washed twice with BSM. Cells were diluted (1:20) in BSM containing SA or long-chain alkanes to culture for 5 or 9 days at 30°C, 200 rpm. The growth curves were shown by OD_600_ values that were measured by BioDrop ultraviolet spectrophotometer (Biochrom, Cambridge, UK).

The residual *n*-alkane (C_32_) was measured by gas chromatography as described previously (44). The degradation rate of *n*-alkane is calculated as follows: Degradation rate (%) = [(Initial *n*-alkane − Residual *n*-alkane)/Initial *n*-alkane] × 100.

### DNA-pull down assay

The assay was performed as previously described with some modifications (45). *A. venetianus* RAG-1 was precultured in LB and subsequently transferred to BSM containing 0.1% (wt/vol) *n*-C_32_. The approach is as described above. When strains grew to the logarithmic stage, cells were collected by centrifugation at 6,000 × *g* for 10 min at 4°C. They were then resuspended in BS/THES buffer [22 mM Tris, 4.4 mM EDTA, 10 mM HEPES, 62 mM NaCl, 5 mM CaCl_2_, 50 mM KCl, 8.9% (wt/vol) sucrose, 12% (wt/vol) glycerol, pH 7.5] and disrupted by sonication on ice. Following centrifugation at 12,000 × *g* for 40 min at 4°C, the supernatant was harvested. In parallel, the biotinylated *almA* gene promoter region was amplified by PCR using primers P*_almA_*-biotin-F/ P*_almA_*-R. The PCR product was purified and incubated with 100 µL of streptavidin magnetic beads (Genscript, Nanjing, China) for 1 h at room temperature. After this, the magnetic beads were washed three times using BS/THES buffer. Then, the supernatant was mixed with the immobilized DNA probe on the magnetic beads and incubated at 4°C for 1 h. Subsequently, the magnetic beads were washed three times with BS/THES buffer containing the 10 µg/mL fragmented genome of RAG-1 and then washed three times with BS/THES buffer. Finally, the DNA-bound proteins were eluted with 1 M NaCl buffer and detected by SDS-PAGE, and the protein band of interest was identified by Liquid chromatography-tandem mass spectrometry.

### qRT-PCR analysis

Total RNA was extracted from strains of the log-phase grown in BSM using RNAprep pure cell/Bacteria Kit (Tiangen, Beijing, China) according to the manufacturer’s instructions, followed by reverse transcription with reverse transcriptase (Vazyme, Nanjing, China). qRT-PCR was performed on a real-time PCR detection system MyiQTM2 (Bio-Rad, Hercules, CA, United States) with a total volume of 20 µL containing 1 µg cDNA, specific primers, and RealStar Fast SYBR qPCR Mix (GenStar, Beijing, China). 16S rRNA gene was used as the internal control, and relative expression levels were calculated based on the 2^−ΔΔCT^ method.

### Measurement of TSS

TSS of the *almA* or *apR1* gene was identified by 5′-rapid amplification of the cDNA end (5′RACE). Total RNA was extracted from RAG-1 grown in BSM containing 0.1% *n*-C_32_ by using an RNAprep pure cell/Bacteria Kit (Tiangen, Beijing, China). The cDNA was obtained by using a cDNA Synthesis Kit (Vazyme, Nanjing, China), followed by was purified with a DNA Purification Kit (Tiangen, Beijing, China). Poly (dG) was added to the cDNA by using a terminal deoxynucleotidyl transferase (Takara, Dalian, China). dG-tailed cDNA was amplified with primers *almA*-Race-1 or *apR1*-Race-1 and the abridged anchor primer (AAP). The PCR products were used as a template for the second PCR with primers *almA*-Race-2 or *apR1*-Race-2 and abridged universal amplification primer (AUAP). The final amplified products were purified by a TIANgel Purification Kit (Tiangen, Beijing, China) and then inserted into a pMD19-T vector (Takara, Dalian, China) for sequencing to identify the TSS. The promoter region of *almA* or *apR1* gene was predicted by Softberry (46).

### Sequence analysis of AlmA and APR1

The homologs of AlmA and APR1 were retrieved from the National Center for Biotechnology Information (NCBI) and references. The neighbor-joining phylogenetic tree of homologs was constructed by using MEGA 7.0 with 1,000 bootstrap replicates (47). MSAs were generated by ClustalW2 (48) and Espript 3.0 (49). The conserved domain was predicted by Interpro (50).

### Protein expression and purification

The open reading frame (ORF) of *apR1* gene was amplified using the RAG-1 genome as a template and then was cloned into plasmid pET-28a (+) and pET-MBP by Gibson assembly to yield pET-28a-*apR1* and pET-MBP-*apR1*. The recombinant plasmid was transferred into *E. coli* BL21 (DE3) and identified by PCR. The positive strain was cultured in LB medium supplemented with 50 mg/mL kanamycin at 37°C. When the OD_600_ reached approximately 0.6–0.8, isopropyl-β-d-thiogalactopyranoside (IPTG) was added to LB medium at a final concentration of 0.2 mM. After incubation for 16–20 h at 16°C, cells were harvested, washed, and disrupted by sonication on ice. The recombinant protein was purified by nickel-nitrilotriacetic acid (Ni-NTA) Beads or dextrin Beads (Smart-Lifesciences, Changzhou, China). The molecular mass of the purified protein was analyzed via SDS-PAGE, and the concentration was determined with the Bicinchoninic Acid method.

### Glutaraldehyde cross-linking

0.1% glutaraldehyde was added to the volume containing 5 µg purified 6 × His-tagged APR1, 50 mM Tris-HCl (pH 8.0), and 10% glycerol (vol/vol). After fixation for 0.5–1 h at 30°C, the SDS-PAGE loading buffer was added to terminate the reaction. The oligomerization state of purified protein was analyzed by SDS-PAGE (51).

### Co-transformation experiments

The P*_apR1_-mcherry* vector was constructed in the same way as the P*_almA_-mcherry*. The *apR1* gene was amplified with primer pair UC-*apR1*-F/UC-*apR1*-R and then inserted into pUC18 by Gibson assembly to obtained pUC18-*apR1*. pUC18-*apR1* and P*_almA_-mcherry* or P*_apR1_-mcherry* were co-transformed into *E. coli* DH5α. Plasmid pUC18 was also co-transformed with P*_almA_-mcherry* or P*_apR1_-mcherry* as the negative control. Cells were cultured in LB medium supplemented with 50 µg/mL kanamycin and 100 µg/mL ampicillin at 37°C, and then samples were collected and washed at different times to determine OD_600_ and fluorescence values. Measurements were made as mentioned above.

### Electrophoretic mobility shift assay

The EMSA experiment was carried out via a nonradioactive method (52). The promoter regions of *almA* and *apR1* gene were amplified with primer pairs P*_almA_*-F/P*_almA_*-R and P*_apR1_*-F/P*_apR1_*-R using the RAG-1 genome as a template, and then PCR products were purified with a DNA Purification Kit (Tiangen, Beijing, China). Approximately 110–140 nM DNA probe was mixed with different amounts of purified APR1 and Bovine Serum Albumin (BSA) in EMSA-binding buffer (5 mM Tris-HCl [pH 7.5], 0.1 mM EDTA, 5 mM MgCl_2_, 10 mM KCl, and 0.2 mM DTT) to a total volume of 20 µL. The reaction system was inculcated at 30℃ for 30 min, and then the EMSA loading buffer was added. The native-PAGE was carried out in 0.5 × Tris-Borate-EDTA (TBE). Finally, the band was stained with Nucleic Acid Dye (GenStar, Beijing, China) and visualized under UV.

### Microscale thermophoresis

The MST assay was performed using a Monolith NT.115 (NanoTemper, Munich, Germany) equipment. NT-647-NHS labeled APR1 (10 µM) was mixed with different amounts of *almA* gene promoter in a reaction buffer (50 mM PBS, pH 7.2–7.4). The data were detected and analyzed by MO. control and MO. Affinity Analysis software, receptively.

### Molecular docking and dynamics simulation

The 3D structure of APR1 was obtained from the AlphaFold Protein Structure Database (53). Molecular docking was carried out by AutoDock 4.2 software (54). After molecular docking, the molecular dynamic (MD) stimulation of APR1-C_32_ complexes was performed using the Gromacs2022.3 software at a static temperature of 300 K and atmospheric pressure (1 Bar) (55). The process of free MD simulation consisted of 5,000,000 steps, the step length was 2 fs, and the total duration was 100 ns.

### Statistical analysis

Values are shown as mean ± SD. Data were analyzed by using the Student’s *t*-test with GraphPad Prism version 8.0.2 (GraphPad Software, San Diego, California) (^*^P < 0.05; ^**^P < 0.01; ^***^P < 0.001; ns, no significant). The diagram of the regulatory mechanism is drawn using Figdraw.

## Data Availability

The GenBank accession numbers of the AlmA and APR1 protein sequences are WP_004877609.1 and WP_004876667.1.

## SUPPLEMENTAL MATERIAL

Supplemental material aem.02050-24-s0001.docx Tables S1 to S3; Figures S1 to S12.

ASM does not own the copyrights to Supplemental Material that may be linked to, or accessed through, an article. The authors have granted ASM a non-exclusive, world-wide license to publish the Supplemental Material files. Please contact the corresponding author directly for reuse.
