# Supplementary material for: Regulation mechanism of the long-chain n-alkane monooxygenase gene almA in Acinetobacter venetianus RAG-1
Source: Appl Environ Microbiol. 2024 Dec 26;91(1):e02050-24. doi: 10.1128/aem.02050-24 (PMC11784139; doi:10.1128/aem.02050-24)
Supplement: Supplemental material — Tables S1 to S3; Figures S1 to S12. [file aem.02050-24-s0001.docx]

**Title: Regulation mechanism of the long-chain *n*-alkane monooxygenase gene *almA* in *Acinetobacter venetianus* RAG-1**

Running title: Regulation of the alkane monooxygenase gene *almA*

Shuai Chen^a,1^, Lu Cao^a,1^, Tianhua Lv^a^, Jia Liu^c^, Ge Gao^a^, Mingchang Li^a^, Liyuan Sun^a^, Wenzhuo Tian^a^, Yutong Tian^a^, Guoqiang Li^a*^, Ting Ma^a,b*^

^a^ Key Laboratory of Molecular Microbiology and Technology, Ministry of Education, College of Life Sciences, Nankai University, Tianjin, China

^b^ Tianjin Engineering Technology Center of Green Manufacturing Biobased Materials, Tianjin, China

c. Tianjin Institute of Industrial Biotechnology, Chinese Academy of Sciences, Tianjin, China

*For correspondence: Guoqiang Li and Ting Ma

E-mail: gqli@nankai.edu.cn; tingma@nankai.edu.cn

Tel. 86-22-23508870; Fax 86-22-23508870

Shuai Chen and Lu Cao contributed equally to this work. Author order was determined on the basis of seniority.

**Table S1** Binding free energy of ligand-receptor complex.

| **Energy component** | **APR1/*n*-C_32_ Complex** |
| --- | --- |
| VDWAALS | -77.31±2.22 |
| EEL | -0.22±0.40 |
| EGB | 12.53±0.65 |
| ESURF | -10.83±0.20 |
| DELTA G gas | -77.53±2.25 |
| DELTA G solv | 1.70±0.77 |
| DELTA TOTAL | -75.83±2.75 |

**Table S2** Hydrocarbon degradation ability of strains in which AlmA/APR1 homologs are present.

| **Strain** | **Substrate** | **References** |
| --- | --- | --- |
| *Acinetobacter venetianus* RAG-1 | C_10_-C_38_ *n*-alkanes | (1) |
| *Acinetobacter oleivorans* DR1 | C_12_-C_38_ *n*-alkanes | (2) |
| *Acinetobacter baylyi* ADP1 | C_26_-C_36_ *n*-alkanes | (3) |
| *Alkanindiges illinoisensis* DSM 15370 | C_16_-C_17_ *n*-alkanes, pristane, qualane | (4) |
| *Alcanivorax borkumensis* SK2 | C_9_-C_32_ *n*-alkanes, phytane, pristane | (5) |
| *Alcanivorax hongdengensis* A-11-3 | C_10_-C_38_ *n*-alkanes, pristane | (6) |
| *Marinobacter aromaticivorans* D15-8P | C_14_-C_20_ *n*-alkanes, naphthalene, phenanthrene and anthracene | (7) |
| *Oleiphilus messinensis* ME102 | C_11_-C_20_ *n*-alkanes | (8) |
| *Ketobacter alkanivorans* GI5 | C_10_–C_28_ *n*-alkanes | (9) |
| *Oleispira antarctica* RB-8 | C_10_–C_24_ *n*-alkanes, pristane | (10) |
| *Alcanivorax dieselolei* B-5 | C_5_–C_36_ *n*-alkanes | (11) |
| *Burkholderia cepacia* RR10 | C_12_-C_30_ *n*-alkanes | (12) |

**Table S3** The amino acid sequence identity of AlmR and APR1.

| **Locus_tag**  **(Gene)** | **Locus_tag**  **(Protein)** | **Protein**  **name** | **organism** | **Sequence**  **identity (%)** |
| --- | --- | --- | --- | --- |
| B5T_02054 | WP_014994400.1 | AlmR | *Alcanivorax dieselolei* B5 |  |
| F959_00251 | WP_004876667.1 | APR1 | *Acinetobacter venetianus* RAG-1 | 4.86% |


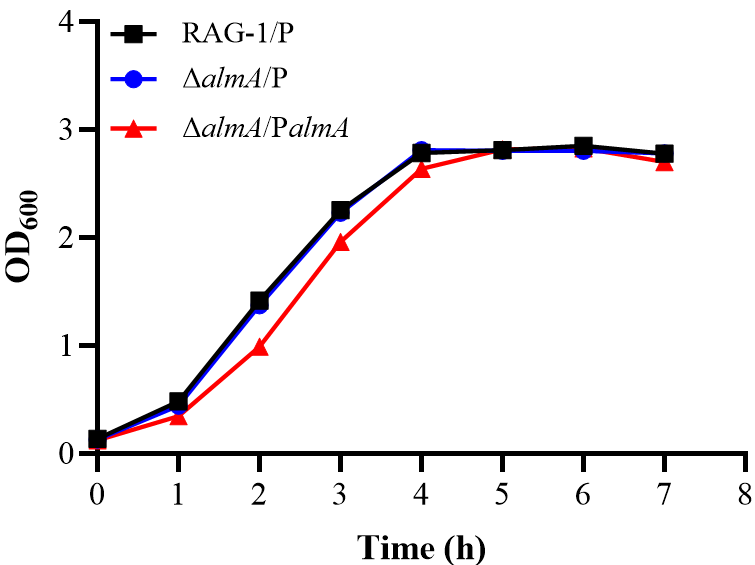


**Fig S1.** Growth curves of WT, Δ*almA*/P, and Δ*almA*/P*almA* strains cultured in LB medium for 7 h.


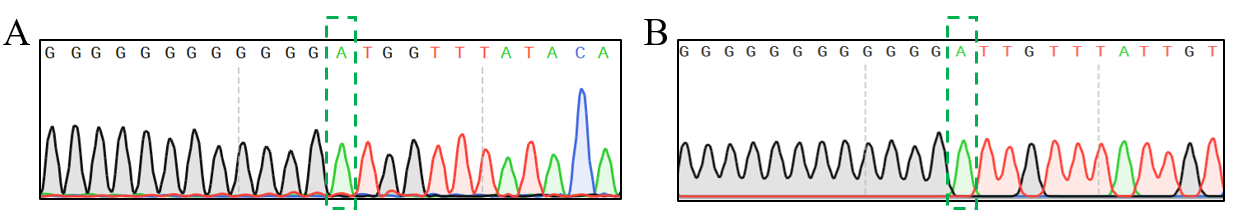


**Fig S2.** TSS determination of the *almA* gene (A) and the *apR1* gene (B) by 5´RACE. The bases circled in green indicate TSSs.


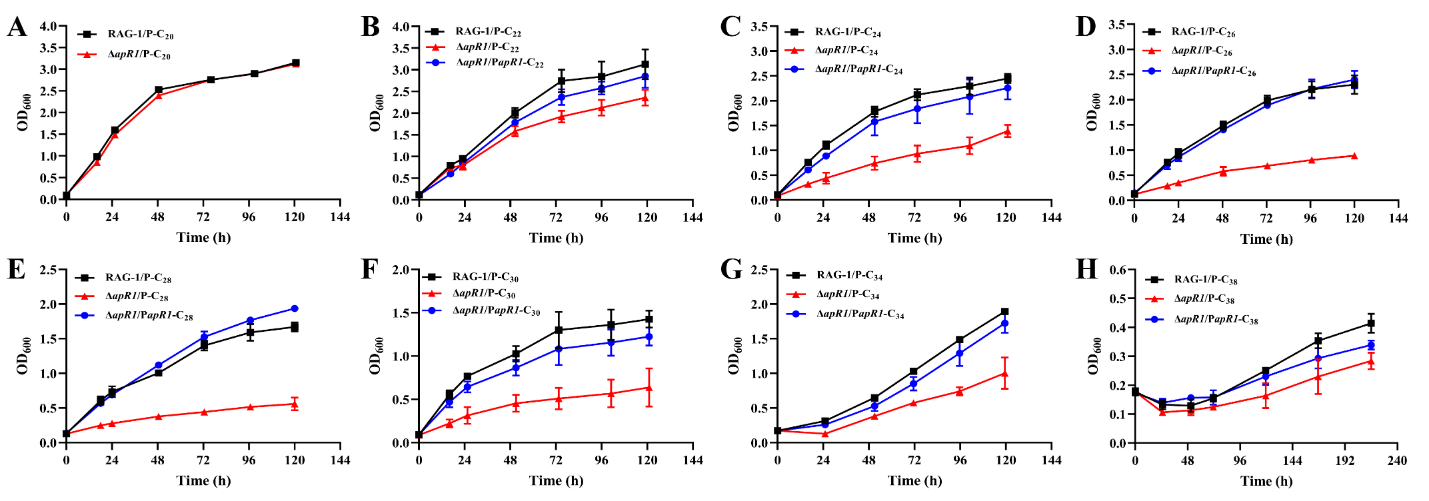


**Fig S3.** Growth of RAG-1/P, △*apR1*/P, and △*apR1*/P*apR1* strains on long-chain *n*-alkanes. These strains are cultured in BSM medium with *n*-C_20_ (A), *n*-C_22_ (B), *n*-C_24_ (C), *n*-C_26_ (D), *n*-C_28_ (E), *n*-C_30_ (F), *n*-C_34_ (G), *n*-C_38_ (H) and as the sole carbon source. Values are shown as mean ± SD (n=3).


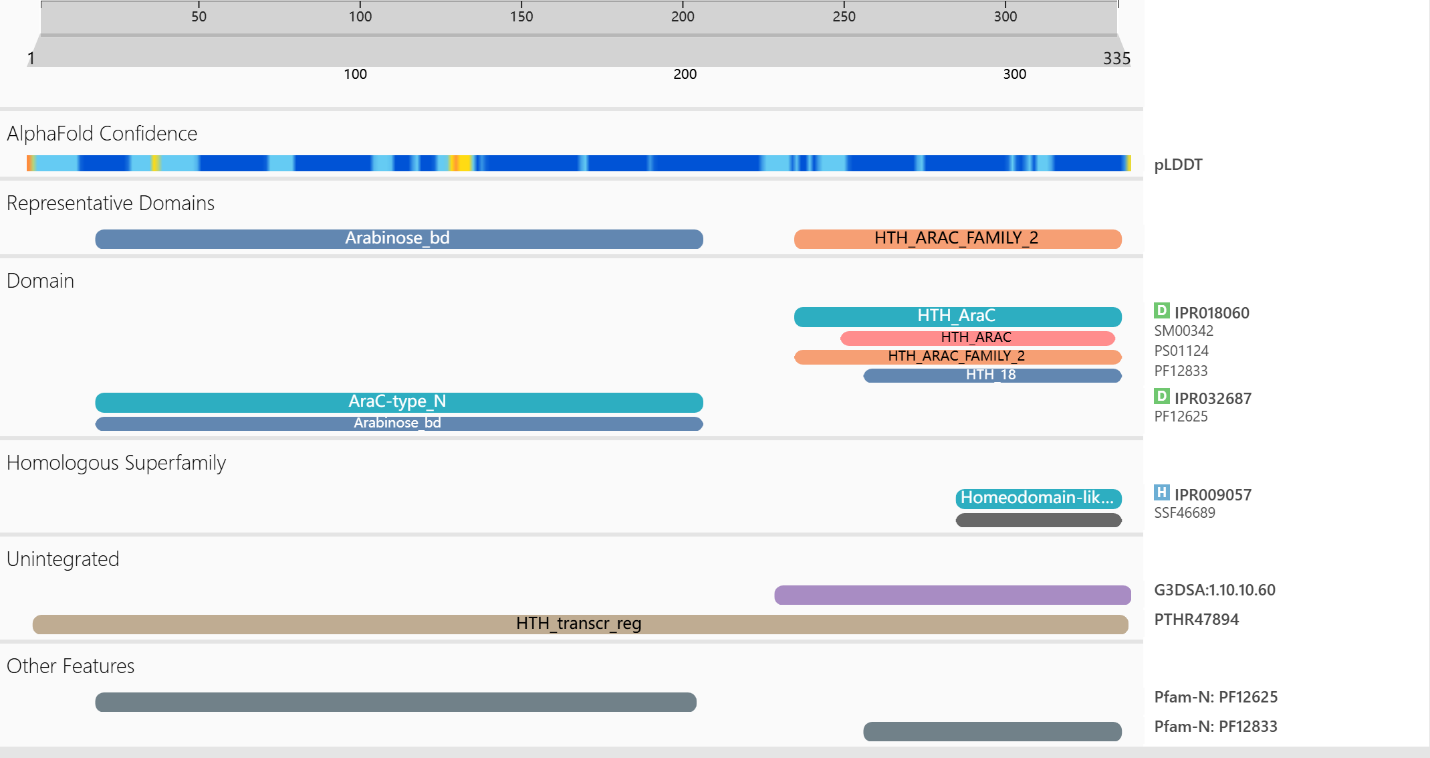


**Fig S4.** Conserved domain analysis of APR1 by InterPro (https://www.ebi.ac.uk/interpro/).

**
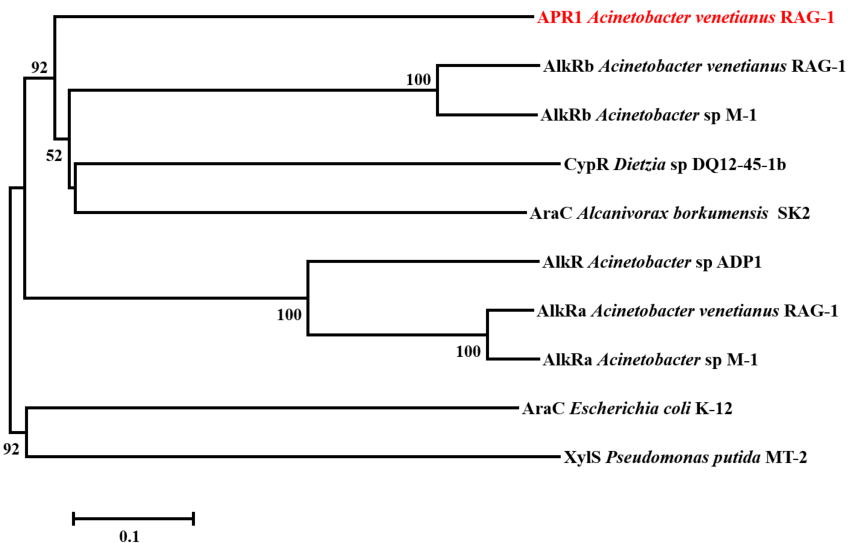
**

**Fig S5.** Phylogenetic analysis of APR1 and other Arac/XylS family transcriptional regulators of hydrocarbon-degrading bacteria by neighbor-joining method (1000 replicates). The APR1 from *A. venetianus* RAG-1 is marked with red. Bootstrap values (%) are indicated at the branch nodes, the scale bar represents 0.1 substitutions per site.


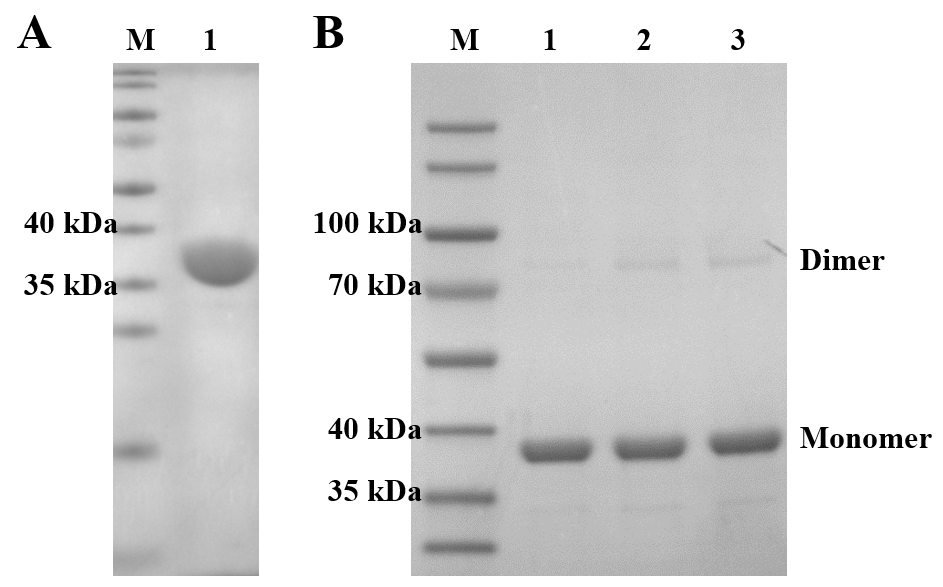


**Fig S6.** SDS-PAGE analysis of the purified 6×His-tagged APR1. (A) The 6×His-tagged APR1 protein was purified by Ni-NTA affinity chromatography. The theoretical size of 6×His-tagged APR1 is 39.2 kDa. Lane M, protein marker; lane 1, purified 6×His-tagged APR1. (B) The oligomerization state of 6×His-tagged APR1 protein was crosslinked with 0.1% glutaraldehyde for 0 (lane 1), 0.5 (lane 2) and, 1 (lane 3) h, respectively. The theoretical size of the dimer is 78.4 kDa. Lane M, protein marker.


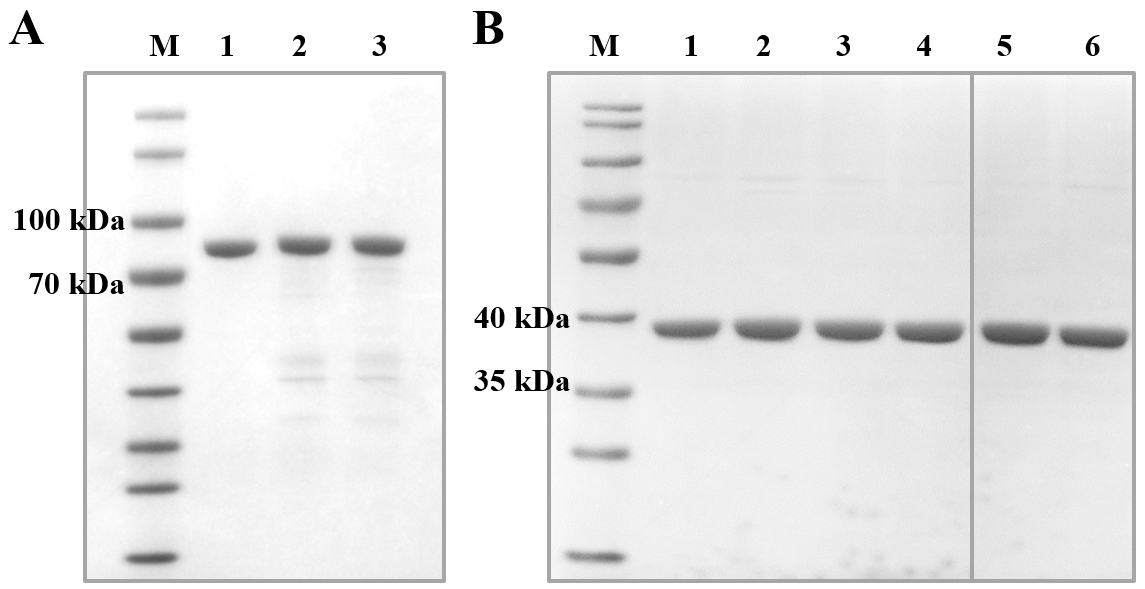


**Fig S7.** SDS-PAGE analysis of the purified APR1. (A) The analysis of the purified MBP-APR1 (lane 1), MBP-APR1^L265S^ (lane 2) and MBP-APR1^L292S^. The theoretical size of MBP-tagged APR1 is 78.3 kDa. (B) The analysis of the purified 6×His-APR1^V303E^ (lane 1), 6×His-APR1^T306L^ (lane 2), 6×His-APR1^F314L^ (lane 3), 6×His-APR1^F318S^ (lane 4), 6×His-APR1^P326S^ (lane 5), 6×His-APR1^R330W^ (lane 6). Lane M, protein marker.


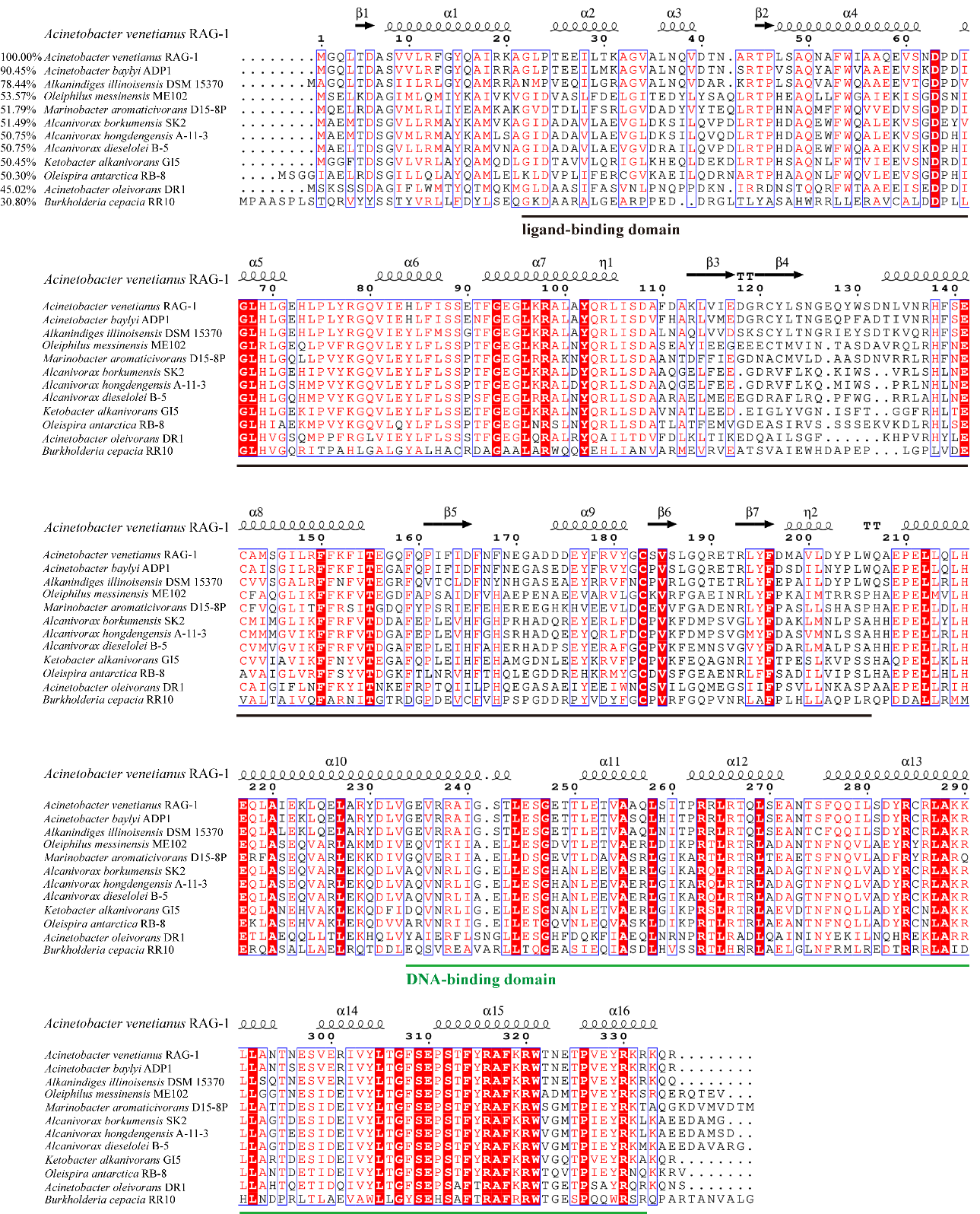


**Fig S8.** Multiple sequence alignments of APR1 and its homologs from different strains in proteobacteria. The secondary structural elements of APR1 are listed above the sequence. The red-filled boxes represent completely conserved sequences in these strains, and the unfilled boxes represent relatively conserved sequences. The ligand-binding domain is shown black underlined and the DNA-binding domain is shown green underlined.

**
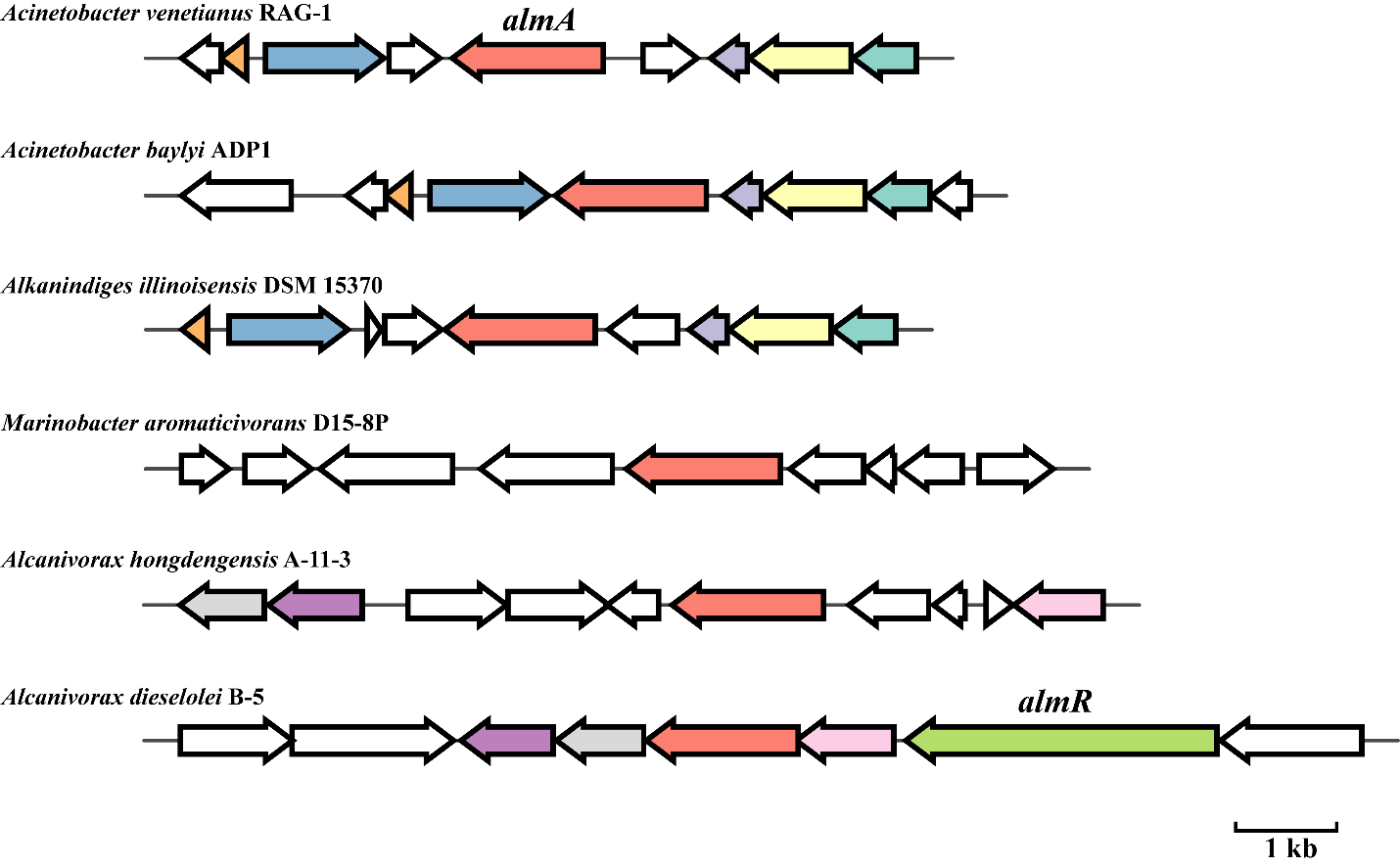
**

**Fig S9.** The organization and composition of *almA* gene cluster from different *n*-alkane-degrading strains. The figure was drawn by Chiplot (https://www.chiplot.online/).

**
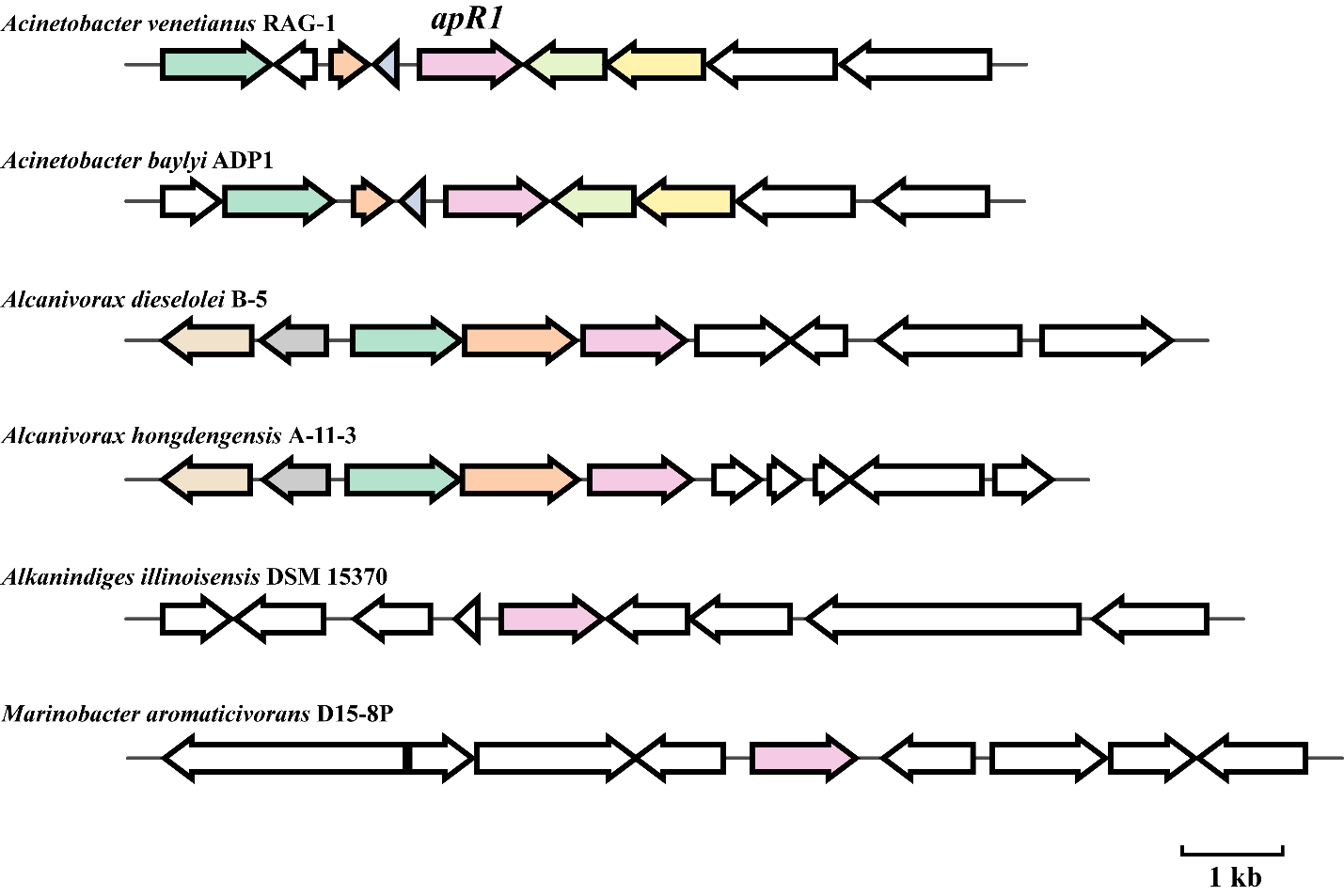
Fig S10.** The organization and composition of *apR1* gene cluster from different *n*-alkane-degrading strains. The figure was drawn by Chiplot.

**
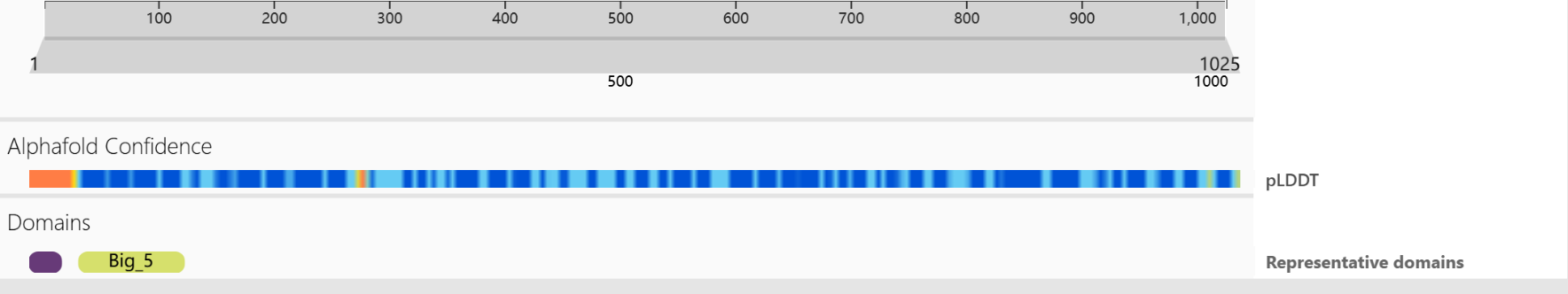
**

**Fig S11.** Conserved domain analysis of AlmR by InterPro.

**
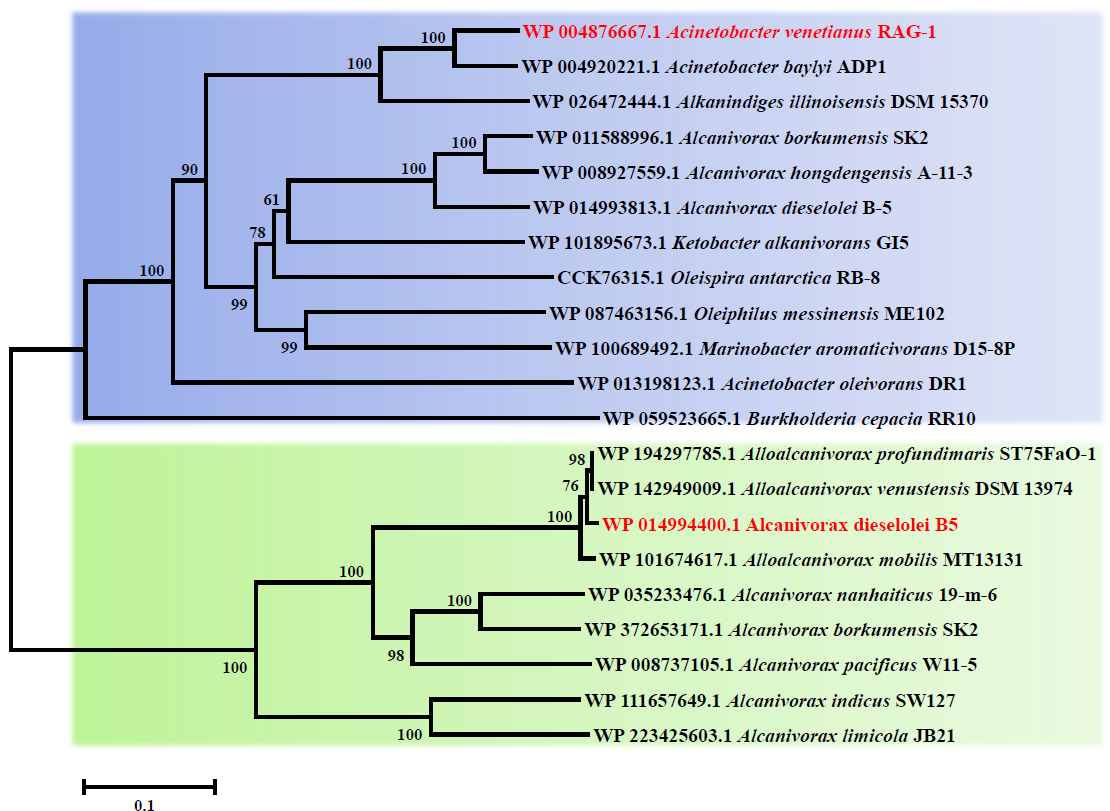
 Fig S12.** Phylogenetic analysis based on the complete amino acid sequences of APR1 homologs and AlmR homologs. The APR1/AlmR homologs are marked with blue/green background. Bootstrap values (%) are indicated at the branch nodes, the scale bar represents 0.1 substitutions per site.

**References**

1. Liu J, Zhao B, Lan YZ, Ma T. 2021. Enhanced degradation of different crude oils by defined engineered consortia of *Acinetobacter venetianus* RAG-1 mutants based on their alkane metabolism. Bioresour Technol 327:124787.

2. Park C, Shin B, Jung J, Lee Y, Park W. 2017. Metabolic and stress responses of *Acinetobacter oleivorans* DR1 during long-chain alkane degradation. Microb Biotechnol 10:1809-1823.

3. Yin CF, Nie Y, Li T, Zhou NY. 2024. AlmA involved in the long-chain *n*-alkane degradation pathway in *Acinetobacter baylyi* ADP1 is a Baeyer-Villiger monooxygenase. Appl Environ Microbiol 90.

4. Bogan BW, Sullivan WR, Kayser KJ, Derr KD, Aldrich HC, Peterek JR. 2003. *Alkanindiges illinoisensis* gen. nov., sp nov., an obligately hydrocarbonoclastic, aerobic squalane-degrading bacterium isolated from oilfield soils. Int J Syst Evol Microbiol 53:1389-1395.

5. Gregson BH, Metodieva G, Metodiev MV, McKew BA. 2019. Differential protein expression during growth on linear versus branched alkanes in the obligate marine hydrocarbon-degrading bacterium *Alcanivorax borkumensis* SK2^T^. Environ Microbiol 21:2347-2359.

6. Wang WP, Shao ZZ. 2012. Genes involved in alkane degradation in the Alcanivorax hongdengensis strain A-11-3. Applied Microbiology and Biotechnology 94:437-448.

7. Cui ZS, Gao W, Xu GF, Luan X, Li Q, Yin XF, Huang DM, Zheng L. 2016. *Marinobacter aromaticivorans* sp nov., a polycyclic aromatic hydrocarbon-degrading bacterium isolated from sea sediment. Int J Syst Evol Microbiol 66:353-359.

8. Toshchakov SV, Korzhenkov AA, Chernikova TN, Ferrer M, Golyshina OV, Yakimov MM, Golyshin PN. 2017. The genome analysis of *Oleiphilus messinensis* ME102 (DSM 13489^T^) reveals backgrounds of its obligate alkane-devouring marine lifestyle. Mar Genomics 36:41-47.

9. Kim SH, Kim JG, Jung MY, Kim SJ, Gwak JH, Yu WJ, Roh SW, Kim YH, Rhee SK. 2018. *Ketobacter alkanivorans* gen. nov., sp nov., an *n*-alkane-degrading bacterium isolated from seawater. Int J Syst Evol Microbiol 68:2258-2264.

10. Gregson BH, Metodieva G, Metodiev MV, Golyshin PN, McKew BA. 2020. Protein expression in the obligate hydrocarbon-degrading psychrophile *Oleispira antarctica* RB-8 during alkane degradation and cold tolerance. Environ Microbiol 22:1870-1883.

11. Wang WP, Shao ZZ. 2014. The long-chain alkane metabolism network of *Alcanivorax dieselolei*. Nat Commun 5:5755.

12. Marín MM, Smits THM, Van Beilen JB, Rojo F. 2001. The alkane hydroxylase gene of *Burkholderia cepacia* RR10 is under catabolite repression control. J Bacteriol 183:4202-4209.
